# The surgical intelligent knife distinguishes normal, borderline and malignant gynaecological tissues using rapid evaporative ionisation mass spectrometry (REIMS)

**DOI:** 10.1038/s41416-018-0048-3

**Published:** 2018-04-19

**Authors:** David L Phelps, Júlia Balog, Louise F Gildea, Zsolt Bodai, Adele Savage, Mona A El-Bahrawy, Abigail VM Speller, Francesca Rosini, Hiromi Kudo, James S McKenzie, Robert Brown, Zoltán Takáts, Sadaf Ghaem-Maghami

**Affiliations:** 1 Imperial College, London, UK; 2 Waters Research Centre, Budapest, Hungary

**Keywords:** Ovarian cancer, Surgical oncology, Metabolomics

## Abstract

**Background:**

Survival from ovarian cancer (OC) is improved with surgery, but surgery can be complex and tumour identification, especially for borderline ovarian tumours (BOT), is challenging. The Rapid Evaporative Ionisation Mass Spectrometric (REIMS) technique reports tissue histology in real-time by analysing aerosolised tissue during electrosurgical dissection.

**Methods:**

Aerosol produced during diathermy of tissues was sampled with the REIMS interface. Histological diagnosis and mass spectra featuring complex lipid species populated a reference database on which principal component, linear discriminant and leave-one-patient-out cross-validation analyses were performed.

**Results:**

A total of 198 patients provided 335 tissue samples, yielding 3384 spectra. Cross-validated OC classification vs separate normal tissues was high (97·4% sensitivity, 100% specificity). BOT were readily distinguishable from OC (sensitivity 90.5%, specificity 89.7%). Validation with fresh tissue lead to excellent OC detection (100% accuracy). Histological agreement between iKnife and histopathologist was very good (kappa 0.84, *P* < 0.001, *z* = 3.3). Five predominantly phosphatidic acid (PA(36:2)) and phosphatidyl-ethanolamine (PE(34:2)) lipid species were identified as being significantly more abundant in OC compared to normal tissue or BOT (*P *< 0.001, *q *< 0.001).

**Conclusions:**

The REIMS iKnife distinguishes gynaecological tissues by analysing mass-spectrometry-derived lipidomes from tissue diathermy aerosols. Rapid intra-operative gynaecological tissue diagnosis may improve surgical care when histology is unknown, leading to personalised operations tailored to the individual.

## INTRODUCTION

Primary epithelial ovarian cancer (OC) has poor prognosis, remains the most lethal gynaecological malignancy and presents with advanced-stage disease in over three-quarters of patients.^1^ Disease burden can be extensive and involve metastatic dissemination to the upper-abdomen, pleura and serosal surfaces of the bowel, liver and spleen. Five-year survival, when presenting at stage three and four, is 39 and 17%, respectively.^2^ Cytoreductive surgery, that renders patients tumour-free, improves prognosis. Three-year overall survival (OS) in patients with zero residual disease after surgery is 72.4 vs 45.2% in patients with >10 mm residual disease.^3–8^ This recognised survival benefit inevitably promotes a radical surgical approach and often includes appendicectomy, splenectomy, peritonectomy and omentectomy, as well as diaphragmatic stripping and total hysterectomy with bilateral salpingo-oophorectomy. Most patients will also receive platinum and taxane-based chemotherapy.

Pre-operatively the nature of the tumour is often unknown, especially if there is no extra-ovarian disease. An attempt at histological diagnosis can be made intra-operatively, but the only established technique, frozen section, is time-consuming and its diagnostic accuracy varies. A meta analysis of frozen section accuracy showed sensitivity to be 65–97% for benign tumours and 71–100% for malignant tumours.^9^ Borderline ovarian tumours (BOT) are especially difficult to characterise.^10–13^ A Cochrane review revealed that BOT diagnosed at frozen section have a 21% chance of finally being reported as OC.^12^ This is significant as BOT rarely metastasise and long-term survival is significantly better than OC.^14^ Women with BOT are more likely to be young and, therefore, may wish to preserve fertility with conservative surgery. The risk of over-treatment in these cases is as great as the risk of under-treatment.^15^

An intra-operative tool that could enable the surgeon to differentiate between BOT and OC would allow the surgeon to tailor the operation to the patient in real-time. The histological diagnosis can significantly alter the course of treatment for the patient, ranging from full clearance of gynaecological organs with full surgical staging for cancer, to a simple unilateral cystectomy for a benign cyst. There are obvious advantages to making a fast, accurate and reliable intra-operative diagnosis. Inaccurate intra-operative diagnosis of BOT results in unnecessarily radical surgery in some, or the need for two procedures in others if OC is ultimately diagnosed. In addition, in the presence of OC that is confined to the ovary without capsular breach, systematic lymph node dissection of pelvic and para-aortic nodes can result in complete staging of the tumour without the need for a second procedure.

Electrosurgical diathermy is used to cut tissue during surgery as it provides haemostasis. Surgical aerosol is a by-product of thermal tissue ablation. We hypothesised that this aerosol was rich in biological information and developed a mass spectrometric method for the online analysis of samples.^16–18^ The use of diathermy as a sampling tool and ion-source, for subsequent mass spectrometric analysis, has been termed Rapid Evaporative Ionisation Mass Spectrometry (REIMS).^17^ REIMS converts molecular constituents into charged gaseous particles (ions), using an ordinary surgical tool, making it ideal for intra-operative use. REIMS employs multivariate statistical analyses to translate spectral data into clinically relevant real-time information. The combination of electrosurgical dissection methods and mass spectrometry (MS) in a single device that provides real-time intra-operative histological information was termed ‘iKnife’. The iKnife has shown sufficient accuracy for ex-vivo identification of liver, lung and colon.^17^ More recently it has shown significant promise in the gastro-intestinal and breast settings successfully distinguishing colorectal carcinoma from normal adjacent mucosa (94.4% accuracy) and breast carcinoma from normal breast tissue (95.8% accuracy).^19,20^ The REIMS iKnife has also identified the origin of metastatic lesions in ex-vivo and in-vivo settings including the endoscopic classification of intestinal wall, cancer and polyps.^21^

Here we present the first use of the REIMS iKnife in the ex-vivo and in-vivo gynaecological settings. We present results obtained from frozen tissue samples and show excellent histopathological discrimination between normal, benign, BOT and OC tissue types. We have validated these results in prospectively collected fresh tissue. In addition, we compare accurate diagnosis of tissue type between the iKnife, surgeon and histopathologist using inter-rater agreement analyses. Furthermore, we evaluate the chemical composition of the tissue classes to identify lipid species that have variable intensity in a range of gynaecological tissue types.

## Materials and Methods

**Fig. 1:**
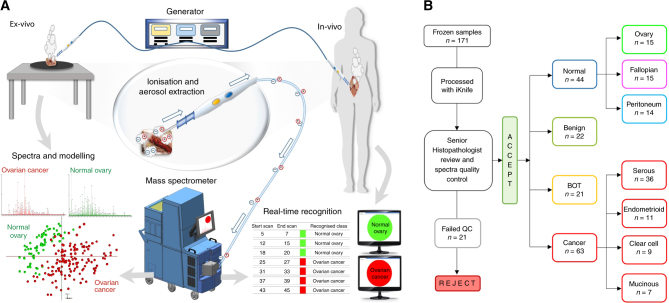
REIMS set-up ex-vivo and in operating theatres (in-vivo) and frozen sample work-flow. **a** Electrical current, produced from the generator, is applied to the tissue and the resultant charged particles are extracted through the custom-designed hand-piece and drawn into the REIMS atmospheric inlet and analysed in the Xevo G2-XS mass spectrometer to produce tissue-specific mass spectra, which are then subjected to multivariate statistical analysis using PC-LDA. Within one to two seconds, real-time tissue diagnosis is displayed on a screen for the surgeon to see. **b** Work-flow for the frozen samples; all samples collected from the frozen tissue bank were processed with the iKnife. After histopathology reporting 22 samples were rejected from the study due to not being gynaecological or epithelial ovarian samples, or the histology was unclear and they failed quality control (QC). The remainder of the samples (*n* = 150) and resultant spectra were included in subsequent models, univariate and multivariate analyses

This prospective observational feasibility study was approved by Imperial College London (ICL) Research and Ethics Committee (REC 14/EE/0024). The handheld surgical diathermy is a standard commercially available device with in-built aerosol extraction tubing. The iKnife system is a non-FDA approved device used for the purpose of research in an investigational setting. Frozen samples were issued by ICL tissue bank between November 2014 and February 2015. Fresh samples were collected and processed between March and November 2016. All the samples were processed at Imperial College Healthcare NHS Trust (ICHNT), London. Our aim was to investigate whether gynaecological tissues yielded unique REIMS signatures and to build a histologically assigned database upon which models could be created to recognise tissue spectra and report histology. Figure 1a summarises the setup and work-flow for this study.

### Sample details

In total, 335 samples (Frozen *n* = 171, Fresh ex-vivo *n* = 119, Fresh in-vivo *n* = 45) were collected from 198 patients (Frozen *n* = 157, Fresh ex-vivo *n* = 35, Fresh in-vivo *n* = 6), which yielded 3384 MS spectra. Some frozen samples were later excluded; 21 samples (12.3%) failed quality control for the following reasons: inconclusive histopathology report (*n* = 8), necrotic tissue (*n* = 5), non-OC (*n* = 6) and technical reasons (histology processing error *n* = 1, no interpretable MS signal *n* = 1). The remaining 150 samples were categorised as ‘normal’ (fallopian tube, normal ovary and peritoneum) (*n* = 44), ‘benign’ (*n* = 22), ‘BOT’ (*n* = 21) or ‘OC’ (*n* = 63) (Fig. 1b). Some patients provided more than one sample, for example a piece of tumour as well as normal fallopian tube or peritoneum. Normal samples of ovary, fallopian tube, peritoneum and omentum were collected from women undergoing gynaecological surgery for benign conditions or stage Ia/b endometrial carcinoma, as the extra-uterine tissues are unaffected by tumour. Benign, BOT and OC tumour samples, including common histological types, were collected from the primary tumour site, except when metastatic tumour was specifically collected. Supplementary table 1 summarises the clinical characteristics of the frozen tumour samples used throughout this work to create the training data set.

### Handling of samples and data

Frozen samples had been snap-frozen and stored at −80°C. Fresh samples (supplementary table 2), kept at room temperature, were processed within 4 h after excision from the patient. Sixteen large samples of omentum and peritoneum (~50 mm in diameter) were collected in order to macroscopically assess the metastatic tumour environment and to enable clinical classification of the tissue. Intra-operative samples were collected during surgery and were obtained by sampling tissue during resection from the patient. Histopathology, sampling site, clinical diagnosis, tumour stage and grade, was recorded on a hospital-networked computer for all samples in the study.

### Processing of samples with REIMS iKnife

Samples were cut (diathermised) multiple times with a modified electrosurgical hand-piece coupled with a Covidien ForceTriad™ generator using 20–25 watts. Multiple unique tissue sampling points (burns) were performed to create surgical smoke (aerosol). The number of unique tissue sampling points was dependent upon the size of the samples. The resultant aerosol was analysed by a modified Waters G2-XS time of flight mass spectrometer in negative-ion sensitivity mode. The REIMS method has been previously described.^16,17,20^ Briefly, the instrument was calibrated daily using sodium formate solution as per the manufacturer’s instructions. Isopropyl alcohol (propan-2-ol) was injected as the solvent matrix at a flow rate of 0.2 ml/min. An external lock mass of leucine enkephalin (1 ng/μl) was used for all fresh tissue work (mass in negative-ion mode 554.2615 *m*/*z*). A known phospholipid (699.497 *m*/*z*) was used for internal lock mass correction for all frozen work. Scan range and time was 50–1200 *m*/*z* and 1 sec, respectively. Fresh samples were processed similarly; however, these were additionally processed in coagulation mode. Sixteen of the fresh ex-vivo samples with tumour metastasis were extensively sampled with multiple sampling sites. Metastatic tumour deposits were sampled directly (positive control), at the lateral edge, and then at increasing distances from the edge of the metastasis. Spectra within the 600–1000 *m*/*z* range were analysed for all samples.

### Histopathological processing and reporting

Processed tissue was formalin-fixed, paraffin-embedded (FFPE), stained with haematoxylin and eosin (H&E) and scanned at high resolution using NDP.view2 software (Hamamatsu Photonics, Japan). The FFPE tissue samples were reported by senior histopathologists (MAEB, AVMS, FR) at Imperial College London to classify the histological environment of the analysis points. Details including tissue type, histology and tumour-cell-content (supplementary figure 1) were provided. The histopathology technician was provided with sketches and photographs of the sixteen large samples to accurately orientate the samples after the process of histological slide preparation. The final histopathological report was used as the gold standard for diagnosis and the tissue sample database was updated accordingly. Inconclusive diagnoses, non-ovarian tumour types and non-epithelial ovarian tumours were excluded from the analyses.

### Statistical analysis and models

Lock mass correction, background subtraction and binning of peaks to 0.1 Da was performed using Waters Corporation’s software (Offline Model Builder (OMB) v1.1.29.0; not commercially available). OMB and MatLab (v2014a) were used for all multivariate analyses including principal component (PCA), linear discriminant analyses (LDA) and loadings plots. Cancer samples were excluded for model building if less than 50% viable tumour was present (supplementary figure 1). For the purposes of model building, the normal tissue class included normal ovary, normal fallopian tube and normal peritoneum in all frozen tissue models. In metastatic samples, the normal class included normal omentum and peritoneum. Validation of each PC-LDA model was performed using leave-one-patient-out cross-validation (LOPOCV), which leaves out all spectra from one patient and builds a model with spectra from all remaining patients. Tissue PC-LDA models created in OMB software were exported into the recognition software to serve as training data sets. OMB Recognition software was used in post-processing mode for characterisation of blind samples.

Univariate analyses were performed using either the Wilcoxon rank-sum or Kruskal–Wallis tests to identify discriminatory ionic species (*P*-values reported). False discovery rate correction (α 0.01) was performed using the Benjamini–Hochberg–Yekutieli method (*q*-values reported).

Cohen’s kappa (κ) inter-rater reliability agreement analyses were performed between three raters of tissue type ((1) Surgeon, (2) iKnife and (3) Histopathologist) on fresh ex-vivo metastatic tissue samples using RStudio (version 3.2.2, https://cran.r-project.org). The surgeon reported their impression of the tissue type at each burn site during sampling with the iKnife. OMB Recognition software was used to give the iKnife’s impression of tissue type at each burn site, using an appropriate tissue PC-LDA model. After histopathological reporting, an agreement analysis between all three reporters was performed. Agreement was categorised by the following κ values: 0.00 no agreement, 0.01–0.20 poor, 0.21–0.40 fair, 0.41–0.60 moderate, 0.61–0.80 good and ≥0.81 very good.

### Lipid identification

LIPID Metabolite and Pathways Strategy (LIPID MAPS®) Lipidomic Gateway (www.lipidmaps.org) was used to provide tentative lipid identification. This was performed for peaks of interest in PC-LDA models, restricting the search to only deprotonated [M – H]^−^ glycerophospholipid and fatty acid ions, with a mass tolerance of +/− 0.1 *m*/*z*, and even-chains only.

REIMS tandem MS (REI-MS/MS) was performed on frozen tissue samples using the Xevo G2-XS Q-Tof instrument. Mass peaks of interest that contributed strongly to class separation in PC-LDA models were selected for fragment ion scan analysis. For these experiments *m*/*z* 699.497, 744.555 and 673.481 were selected as the ‘MS/MS mass’. Tissue samples were processed in cut mode, 20 W, using Argon as the collision gas. Tissue sampling was performed over 5 sec to gain adequate volumes of aerosol for MS/MS analysis. MS/MS negative-ion-mode spectra were presented to the LIPID MAPS online tool with the following parameters: intensity threshold 5, ion mass tolerance +/− 0.1 *m*/*z*, any head-group.

## Results

### Statistical modelling with frozen samples—normal vs cancer tissue

**Fig. 2:**
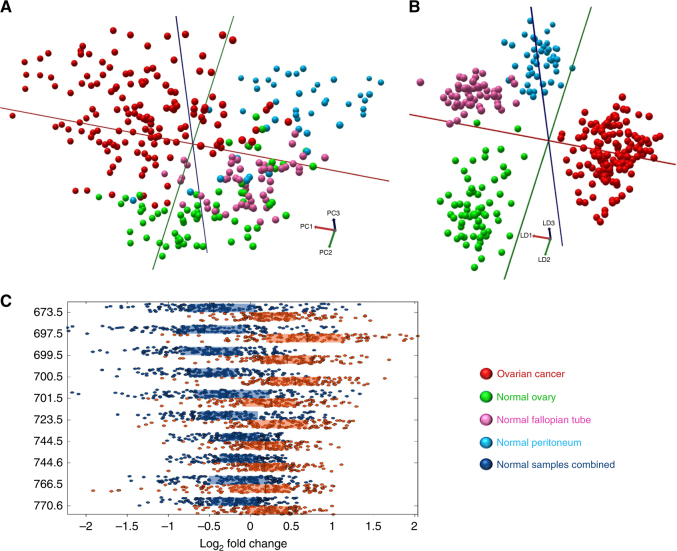
Multivariate analyses of ovarian cancer and normal tissue types. **a** 3-component 3D PCA, percentage of variance explained in PCs1–3: 36.0, 17.6, 9.0%. **b** 25-component 3D PC-LDA. **c** Box plots of univariate analysis showing the top *m*/*z* peaks contributing to class separation (log_2_ fold change), between ovarian cancer and normal samples combined. Ten most significant and intense REIMS spectra peaks shown with *P* < 0.001, *q* < 0.001. Figure based on the following sample numbers: ovarian cancer *n* = 39, normal ovary *n* = 15, normal fallopian tube *n* = 15, normal peritoneum *n* = 14

To establish the iKnife’s ability to distinguish normal gynaecological tissue from OC, a multivariate statistical model was created from the frozen sample spectra, including normal sample types and OC (Fig. 2). Some OC samples had very-little tumour cell content and, therefore, samples with <50% tumour were excluded from the analysis (see supplementary figure 1 for examples), to ensure that mass spectra obtained from the samples were representative of tumour rather than tumour associated stroma.

The OC class separated from normal gynaecological samples along the first principal component (PC1) (Fig. 2a). This suggests that normal gynaecological samples have a significantly different mass spectral profile from OC samples. Derived PCs were used as input variables for PC-LDA (Fig. 2b). Normal ovary and normal peritoneum classes predominantly separate along the second linear discriminant (LD2), while fallopian tube clusters away from the other classes along LD3. Although the separation of the classes is obvious in the PCA plots, further statistical validation of these class separations was performed using LOPOCV. Cross-validated correct classification in this model was 97.6% with OC correct classification sensitivity of 97.4% and specificity of 100% (Supplementary Table 3). In the same analysis, normal fallopian tube and peritoneum were both classified with 100% accuracy. One sample of normal ovary (6.7%) was misclassified as fallopian tube. One OC sample (2.6%) was misclassified as peritoneum, which could be a true misclassification or a result of the iKnife sampling a fibrotic (peritoneum-like) area of the tumour. Univariate analysis was performed to determine, which mass spectral peaks varied significantly between classes. Figure 2c shows the most discriminating top ten significant and abundant *m*/*z* peaks and the relative log2 fold change between tissue classes. Overall the results show that the iKnife can achieve highly accurate classification rates (93–100%) in a model comparing normal gynaecological tissues with OC. However, in clinical practice the distinction between normal tissue and OC is not often a challenge. Further work focused on the ability to distinguish BOT from OC.

### Statistical modelling with frozen samples—invasive and non-invasive tumours

**Fig. 3:**
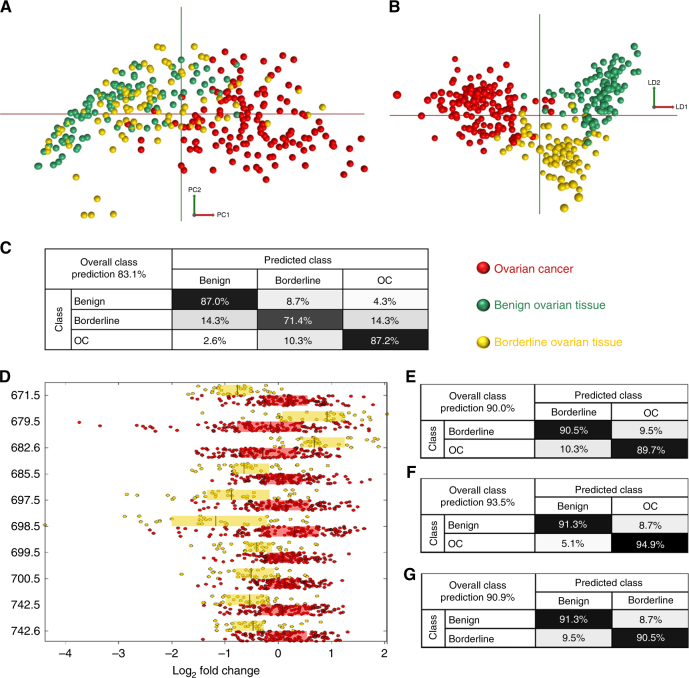
Multivariate statistical analyses of ovarian cancer, benign and borderline ovarian tumours. **a** 2-component PCA, **b** 25-component 2D PC-LDA, **c** Leave-one-patient-out cross-validation when all three tissue classes are included in the model, **d** Box plots of univariate analysis showing the top *m*/*z* peaks contributing to class separation (log_2_ fold change), between ovarian cancer and borderline ovarian tumours. Ten most significant and intense REIMS spectra peaks shown with *P* < 0.001, *q* < 0.001, **e**–**g** Binary LOPOCV models showing improved classification when only two classes are included in the model. Number of samples: ovarian cancer *n* = 39, benign *n* = 22, borderline tumour *n* = 21. Only OC samples with at least 50% of viable tumour were included

The most challenging intra-operative differentiation is between OC and BOT as macroscopically they can appear very similar and intra-operative frozen section diagnostic sensitivity can be as low as 25% for BOT.^13^ To investigate whether non-invasive ovarian tumours (benign and BOT) have differential mass spectral lipidomes from OC, a multivariate PC-LDA model was constructed to compare these tissue classes (Fig. 3).

In PCA analysis there is an obvious grouping of OC away from the benign and BOT samples, particularly along PC1 (Fig. 3a). PC1 always explains the largest variance, which, therefore, suggests that OC, as a distinct class, has a significantly differential lipidomic signature to both benign and BOT samples. This is an important finding, as it is this distinct group of OC patients that require full cancer staging surgery, rather than patients with benign tumours or BOT. In PCA there is little separation between the two non-OC classes. Clear separation can, however, be seen in the LDA model (Fig. 3b). In LDA OC separates from the other classes along the first linear discriminant, with benign and borderline classes separating along LD2. LOPOCV in the model that included all three tissue classes (Fig. 3c) had overall correct classification of 83.1%, with the highest correct classification observed for OC and benign (≥87%). BOT had the lowest correct classification accuracy (71.4%), most likely due to them having similarities to both benign and OC tumours, whereas OC and benign tumours are histologically, biologically and phenotypically the most dissimilar. Univariate analysis was performed to determine, which mass spectral peaks varied significantly between classes. Figure 3d shows the most discriminating top ten significant and abundant *m*/*z* peaks and the relative log2 fold change between OC and BOT tissue classes. To explore whether binary classification models affect the correct classification accuracy, three binary models were created to compare only two classes at any one time. Figure 3e–g show that classification accuracies can be improved significantly when using binary models, with overall correct tissue classifications ranging from 90.0 to 93.5%. OC discrimination from BOT showed high accuracy with sensitivity of 90.5% and specificity of 89.7%.

To determine the effect of freeze–thaw on the samples and to validate the results previously shown, we prospectively collected fresh tissue samples to serve as a validation data set.

### Validation of frozen data sets with prospectively collected fresh samples

**Fig. 4:**
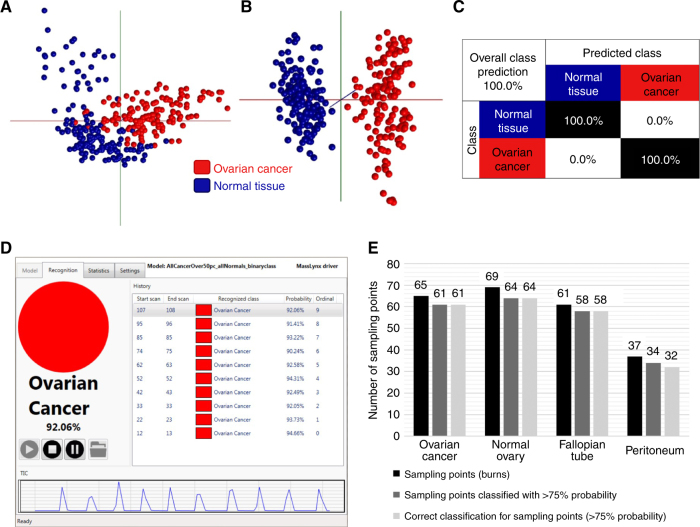
Validation of the frozen tissue model using fresh tissue. **a** 2D PCA model of frozen ovarian cancer samples (>50% tumour content) and all normal samples combined as ‘Normal Tissue’. **b** 3D LDA component analysis. **c** Leave-one-patient-out cross-validation of the binary ‘Ovarian Cancer’ vs ‘Normal Tissue’ frozen model, showing 100% correct classification. **d** OMB Recognition software example showing reported classification for individual sampling points (burns) and the probability value associated with the reported class. **e** Bar chart showing the numbers of sampling points in fresh tissue classified with >75% probability using the OMB recognition software, plus the diagnostic accuracy at those sampling points [OC 100% (61/61), normal ovary 100% (64/64), fallopian tube 100% (58/58), peritoneum 94.1% (32/34)]

An intra-operative tool that could provide a binary classification of ‘Normal’ or ‘Cancer’ would be more useful than a tool which differentiates between fallopian tube and peritoneum for example. A frozen model was, therefore, created to classify new blind fresh samples as ‘Normal Tissue’ or ‘Ovarian Cancer’ (Fig. 4a, b). This model was constructed to check the feasibility of using frozen tissue data as a reference data set for real-time analysis and to serve as a fresh validation of the frozen model. Thirty-five patients donated 119 fresh samples, yielding 2134 unique sampling points with the iKnife (supplementary table 2). Fresh samples of primary OC, normal ovary, fallopian tube and peritoneum totalled 64 of these samples and 32 were processed in cut mode.

The frozen tissue binary model had an internally cross-validated sensitivity and specificity of 100% (Fig. 4c). Each fresh tissue spectrum (burn) was presented to the model using OMB Recognition software (v1.1.29.0) to output, in real-time, the tissue classification (Fig. 4d). OMB Recognition software allows a classification accuracy or reliability threshold to be set. This ensures that all software outputted classifications are deemed, by the software, to be the most reliable. We set this level at 75%, therefore any outputted classifications with a reliability score of <75% were ignored. A total of 232 sampling points (burns) were collected from 32 samples to validate the frozen model with fresh samples (Fig. 4e). Of the 232 sampling points, 217 (93.6%) were reported with >75% reliability. Overall, 215/217 sampling points were correctly classified using the REIMS iKnife, by analysing the tissue lipidome signatures, when compared to gold-standard histology, an overall correct classification rate of 99.1%. Individual OC, normal ovary and fallopian tube sampling points were all classified with 100% accuracy when using the real-time recognition model. There was a 5.9% false positive rate for peritoneal samples, which may be compounded by the smaller number of samples in this class. Supplementary Table 4 summarises the fresh samples processed in this analysis, and the processing points included, showing the associated diagnostic accuracy using the recognition software.

### Comparison of iKnife, surgeon and histopathologist tissue diagnosis in metastasis

**Fig. 5:**
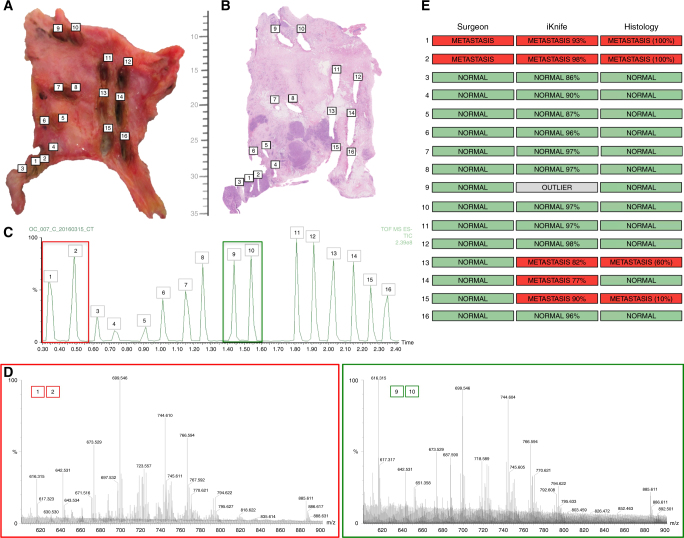
Spatial resolution of metastatic peritoneal deposits and iKnife recognition. **a** Peritoneum containing metastatic tumour deposits with iKnife burns labelled 1–16 sampling normal peritoneum and tumour nodules. **b** Matched haematoxylin and eosin histological slide for the tissue sample in panel A with corresponding burn sites numbered. Scale showing millimetres. **c** Total ion chromatogram obtained during the sampling of the specimen with the iKnife with each burn numbered. Coloured boxes represent data obtained from a nodule (red; burns 1&2) and normal peritoneum (green; burns 9&10). **d** Representative mass spectra, obtained in negative-ion mode from a nodule (red) and normal peritoneum (green) showing the degree of variability in the MS peaks for different tissue types (OC vs normal). **e** Surgeon’s histological impression, iKnife’s impression (percentages in parenthesis represent probability of correct classification) and the histopathologist’s final diagnosis (percentages in parenthesis represent tumour cell content)

Intra-operatively, surgeons rely on tissue characteristics, such as tissue architecture, colour and consistency to form an opinion as to its pathological nature. All previous samples presented in this research paper were relatively small (3–8 mm). This hindered the macroscopic study of the samples to be able to form clinical impressions about tissue type. Figure 5a shows how multiple processing points were possible on the sixteen larger samples. The surgeon planned each sampling point and noted their impression for all sampling points before sampling with the iKnife (Fig. 5e ‘Surgeon’).

Figure 5b shows the matched H&E slide for the tissue processed in Fig. 5a. The sampling points were identified on the total ion chromatogram and resultant mass spectra compared (Fig. 5c, d). Mass spectral peaks at *m*/*z* 699 and *m*/*z* 744 featured amongst the most abundant peaks in both normal and metastatic tissue. The intensity of these two peaks is closely matched in normal tissue, which contrasts with OC where *m*/*z* 699 is significantly higher (Fig. 5d). OMB Recognition software reported iKnife classification for each of the sampling points (Fig. 5e ‘iKnife’). Post-processing histopathological impression is shown in Fig. 5e ‘Histology’. The inter-rater Cohen’s kappa (κ) for the surgeon’s impression compared to the histopathologist was 0.60 (moderate), *P* = 0.0088, *z* = 2.62. Cohen’s κ inter-rater reliability for the iKnife and histopathology was 0.84 (very good), *P* < 0.001, *z* = 3.3. Metastatic deposits could be easily palpated at sampling points 1&2 and all three raters identified this as tumour. All other sampling points appeared normal to the surgeon, but it is clear from Fig. 5b that points 13 and 15 sampled areas of tumour (stained purple), which were not seen by the surgeon, but were identified by the iKnife and the histopathologist. Point 14 on Fig. 5a, b, which is close to other metastatic deposits is reported as normal by the histopathologist, but as metastasis by the iKnife.

In total, 16 metastatic samples were collected, totalling 150 sampling points. All 150 sampling points were processed in the same manner as the sample in Fig. 5 (Supplementary table 5). Unfortunately, the majority of the samples were rare tissue types (mucinous carcinoma and carcinosarcoma), which are not well represented in the cancer model. Therefore, it was not appropriate to perform kappa analysis on these samples.

### Intra-operative in-vivo experiences with iKnife

Promising intra-operative in-vivo results have been observed, providing 119 spectra from 45 tissue samples (*n* = 6 patients) in both cut and coagulation mode. The iKnife has reliably provided high quality mass spectra in the in-vivo setting and data collection continues to enable robust analyses.

### Lipids involved in class separation

Multivariate PC-LDA models perform well in LOPOCV and will be the cornerstone of intra-operative iKnife recognition models. However, these models do not define the lipids responsible for class separation—they merely serve as a tissue fingerprint. LipidMAPS was used to propose deprotonated [M − H]^−^ and chlorinated [M + Cl]^−^ negative ions using the inputted *m*/*z* values from the models. It is also known, from previous work within the iKnife research team, that the REIMS method can result in the deammoniated anion [M − NH_3_]^−^,^20^ but these are not available in LipidMAPS.

The three most significant MS peaks that contribute to the separation of OC from all normal samples are *m*/*z* 673.481, 699.497 and 744.555. *m*/*z* 673.481 likely represents the deprotonated ([M − H]^−^) form of phosphatidic acid (PA(34:1)). [M − H]^−^ ion of phosphatidic acid (PA(36:2)) and [M − NH_3_]^−^ form of phosphatidyl-ethanolamine (PE(34:1)) are the most likely contributors to the peak at *m*/*z* 699.497. [M − H]^−^ phosphatidyl-ethanolamine (PE(36:1)) was tentatively associated with *m*/*z* 744.555. MS peaks at 699.497 *m*/*z* also featured in the OC/BOT model, with peak 699.497 significantly higher in OC than BOT (Fig. 3d). Intensity of the ion associated with deprotonated phosphatidyl-ethanolamine (*m*/*z* 742.539, PE(36:2)) is significantly higher in OC than BOT and [M − H]^−^ phosphatidic-acid-plasmalogen (*m*/*z* 685.517, PA(P-36:1)) is more abundant in OC. The levels of significance, relative arbitrary intensity and median log2 fold change of all highly significant (*p* < 0.001, *q* < 0.001) *m*/*z* peaks contributing to class separation for OC/Normal and OC/BOT are shown in supplementary tables 6 and 7.

To further confirm the tentative lipid assignments, tandem MS was performed on frozen samples of OC, normal ovary, normal peritoneum and normal fallopian tube to confirm the lipid identification for 673.481, 699.497 and 744.555 *m*/*z*. Tandem MS confirmed the tentative assignments listed in Table 1 for the OC/Normal Tissue and OC/BOT models.

**Table 1:** Tentative lipid assignments for the top three significant lipids in each model

Model	*m*/*z*	Lipid	Configuration	Ion
Cancer vs Normal	673.481	Phosphatidic acid	PA(34:1)	[M − H]^−^
	699.497	Phosphatidic acid/Phosphatidyl-ethanolamine	PA(36:2 / PE(34:1)	[M − H]^–^/[M − NH3]^−^
	744.555	Phosphatidyl-ethanolamine	PE(36:1)	[M − H]^−^
Cancer vs BOT	699.497	Phosphatidic acid/Phosphatidyl-ethanolamine	PA(36:2 / PE(34:1)	[M − H]^−^/[M − NH3]^−^
	742.539	Phosphatidyl-ethanolamine	PE(36:2)	[M – H]^−^
	685.517	Phosphatidic-acid-plasmalogen	PA(P-36:1)	[M − H]^−^

Tandem MS and LipidMAPS used along with locally derived lipid data sets from previous REIMS work to tentatively assign lipid classifications and configurations. *PA* phosphatidic acid, *PE* phosphatidyl-ethanolamine

## Discussion

We present the first published report of REIMS characterising gynaecological tissue types in real-time. The robust differentiation of normal tissue types from OC was remarkable (Fig. 4c) with 100% sensitivity and specificity. The REIMS technology provides a close-to-ideal solution as it gives real-time feedback, which can inform the surgeon of accurate histopathological information, allowing them to tailor surgery. Whilst the REIMS technique currently requires tissue destruction with diathermy, it performs better than current non-destructive MS-based tissue identification methods for OC.^22^

Tissue discrimination in the OC and BOT model is promising as it is well recognised that BOT are difficult to diagnose, especially intra-operatively.^9–13^ Whilst these numbers are relatively small, and some caution should be exercised when drawing conclusions, the correct classification results (90.5% sensitivity and 89.7% specificity) are encouraging and could be a progressive development for younger women having treatment for BOT wishing to preserve their fertility. In addition, intra-operative diagnosis of OC confined to the ovary with no capsular breach, will allow removal of lymph nodes for full staging. We recognise that an intra-operative tool, which uses histopathology to guide surgeons towards more personalised management, may have wide-reaching benefits to patients and the health service. We appreciate however, that the number of BOT in this study are relatively low and we endeavour to focus on this particular histology to further improve our model for future testing. Reducing the numbers of tissue classes in the multivariate models appears to increase the correct tissue classification accuracy. This is likely to be as a result of removing a class of tissue that is lipidomically similar. This is supported by the superior classification of OC in Fig. 3f when compared to benign tumours, as these tissue classes are biologically more dissimilar than OC and BOT for example. It could be argued that using binary models introduces bias, as the correct diagnosis is more likely to be made by chance. However, the correct classification rates in the 3-class model (Fig. 3c) rival existing intra-operative diagnostic methods and provide a result in 1.8 sec^20^ rather than 30 to 45 min for frozen section.

The diagnostic tissue accuracy seen in the frozen tissue models was impressive, but caution should be exercised as these samples were processed and analysed retrospectively and the samples were frozen. However, our real-time fresh ex-vivo work corroborated the frozen data. Real-time diagnosis of fresh tissue types using a frozen model was reliable and robust with 217 out of 232 sampling points (93.5%) reported with a classification accuracy of ≥75%. Of the 217 sampling points reported, 215 (99.1%) were correctly diagnosed. This suggests that the phospholipid composition of tissues is not significantly altered by a single freeze–thaw cycle. This is an encouraging finding as it implies that models could be built from historical fresh-frozen tissue bio-banks, which would be especially useful for the much rarer tissue types. Furthermore, use of historical samples would also allow the testing of the prognostic capability of the method, as long-term follow-up data would be available on large numbers of historical samples. The instrument continues to be used intra-operatively (in-vivo) and the acquisition of data is ongoing to build histopathological-spectral libraries. Future work will encompass tissue processing in a clinical trial setting to robustly test the iKnife’s discriminatory ability and to establish whether the improved accuracy of tissue detection may have a positive effect on progression-free and OS.

The larger metastatic tissue samples provide further fresh tissue validation of the frozen models. The purpose of these experiments was to investigate any peri-tumoural halo effect that may be present around each of the metastatic deposits. We hypothesised that normal tissue adjacent to metastatic nodules would display an altered lipidome and be recognised by the OMB Recognition software as tumour. This did not appear to be the case and likely represents the fact that ovarian metastases are known to develop via a trans-coelomic route. OC single cells or spheroids, carried in the peritoneal fluid from the primary tumour, are known to seed onto the peritoneal surface and grow locally rather than invading along its surface.^23,24^ These experiments did, however, present an opportunity to compare the diagnostic ability of surgeons and the iKnife, using histopathology as the gold standard. When comparing Fig. 5a, b it is evident that some tumour deposits seen in the H&E image are not visible to the naked eye. This explains why the iKnife performed better than the surgeon for this sample. It is an interesting finding as it that raises the possibility that surgeons may leave behind tissue that appears normal and the iKnife could be used as a surgical adjunct to test for hidden metastases, or to guide the extent of peritoneal stripping. Point 14 in Fig. 5a, b is notable as the iKnife confidently classified the tissue as metastasis, yet the histopathologist reported normal tissue. This highlights a limitation when validating the iKnife technology—sampling is destructive, therefore limiting histopathological confirmation. Point 14 may very well have been a small focus of tumour, explaining the iKnife’s report, but the tissue no longer exists for it to be confirmed by the histopathologist. The diagnostic ability of the iKnife was hindered, when analysing the 16 large metastatic samples, by the fact that many of the histologies within these samples were rarer (mucinous, carcinosarcoma), and, therefore, did not have significant representation in the iKnife reference database. This highlights a limitation of the iKnife technology; the recognition accuracy will always be impaired when few histologically similar samples exist in the data set. This underlines the need for ongoing data collection to populate the iKnife’s histology and spectra reference database.

An important hallmark of cancer is uncontrolled cellular proliferation, requiring an increased rate of biological-membrane-synthesis that is orders-of-magnitude higher than healthy tissue. Consequently, phospholipid biosynthesis pathways differ between healthy and malignant tissues. Cancer cell metabolism encourages de-novo lipid synthesis of fatty acids by anaerobic glycolysis producing energy and pyruvate (Warburg effect). Fatty acids are incorporated into complex lipids through phosphatidic acids (PAs) as intermediary metabolites. PAs also serve as precursors for phospholipids generated de-novo, or from existing phospholipids, and can serve as cell survival signalling molecules acting primarily on the mammalian target of rapamycin (mTOR).^25^ We observed significantly elevated PA levels in OC tissue using REIMS, which supports its previously established role in phospholipid metabolism and cancer. Whilst we do not currently believe that individual lipids can be used as diagnostic biomarkers alone, when combined they do appear to have a role to play as part of a diagnostic signature. Phospholipids have long been understood to have a role in carcinogenesis and metastasis and have been used to improve diagnostic accuracy in OC.^26,27^ More recently lysophosphatidic acid (LPA), a precursor of PA, and phospholipases involved in the hydroxylation of LPA precursors have all been shown to be abundant in OC.^28–30^ LPA is a well-recognised mitogen and has been shown to stimulate proliferation of OC cell lines.^31^ Furthermore, it is a potent modulator of gene expression, specifically those involved with inflammation, angiogenesis and carcinogenesis, including vascular endothelial growth factor.^32^ We have shown that using REIMS we can identify phospholipid species predominantly within classes of PA and PE in ovarian samples, which our group previously showed in breast tissue.^20^ These findings are further supported by the identification of abundant PA(36:2) and similar PE classes using desorption electrospray ionisation MS (DESI-MS) on OC tissue samples in our group.^33^ The differential intensity of PA species suggests that future REIMS models could focus on these species for more robust diagnostic performance. Further work is planned to identify the species detected throughout this study and to investigate the expression patterns of corresponding genes, which will enable a more targeted approach towards detection of phospholipid biomarkers by REIMS.

REIMS may have the potential to augment surgical decision making in real-time by revolutionising intra-operative histological diagnosis for OC. Tumours that radiologically and visually appear less aggressive, which often receive conservative resection, are occasionally high-grade invasive OC, requiring return to theatre for staging and resection. The iKnife could end this intra-operative uncertainty by providing a rapid and reliable point of care tissue diagnosis. Similarly younger women, wishing to retain fertility, could be assured of real-time accurate diagnosis to guide the surgeon towards the least radical resection for benign or BOT. Furthermore, detection of metastatic tumour deposits, which are not visible to the naked eye, could potentially introduce a new paradigm shift in the treatment of OC by removing microscopic tumour deposits, which may reduce recurrence and improve survival. The current technology is able to use the entire tissue-derived MS-spectrum as a fingerprint to identify the tissues rapidly. The reference data set created throughout this research will be the foundation for future real-time intra-operative tissue diagnosis as part of a clinical trial. New iKnife features and methods are currently in development. The laparoscopic iKnife, for example, would allow less-invasive sampling pre-debulking surgery. Intra-operative REIMS imaging will potentially provide real-time lipid profile mass spectral images of the tissue without the need for diathermy. This non-destructive method could allow more accurate resection of metastatic disease. These exciting iKnife applications should be investigated within appropriately controlled multi-centre clinical trials.

### Availability of data and material

All data included in this manuscript are stored electronically at Imperial College London and access to this can be requested from D.P., S.G.M. or Z.T. by contacting the corresponding authors.

## Electronic supplementary material

Supplementary Figure 1: Examples of varying tumour-cell content in serous adenocarcinoma tumours

Supplementary Table 1: Characteristics of all frozen tumour samples

Supplementary table 2: Fresh samples collected and processed with the iKnife. Patients n=35, samples n=119

Supplementary Table 3: Leave one patient out cross-validated classification of ovarian cancer and normal tissues

Supplementary Table 4:

Supplementary Table 5:

Supplementary Table 6

Supplementary Table 7
