# Supplementary figures and images for: The surgical intelligent knife distinguishes normal, borderline and malignant gynaecological tissues using rapid evaporative ionisation mass spectrometry (REIMS)
Source: Br J Cancer. 2018 Apr 19;118(10):1349–58. doi: 10.1038/s41416-018-0048-3 (PMC5959892; doi:10.1038/s41416-018-0048-3)

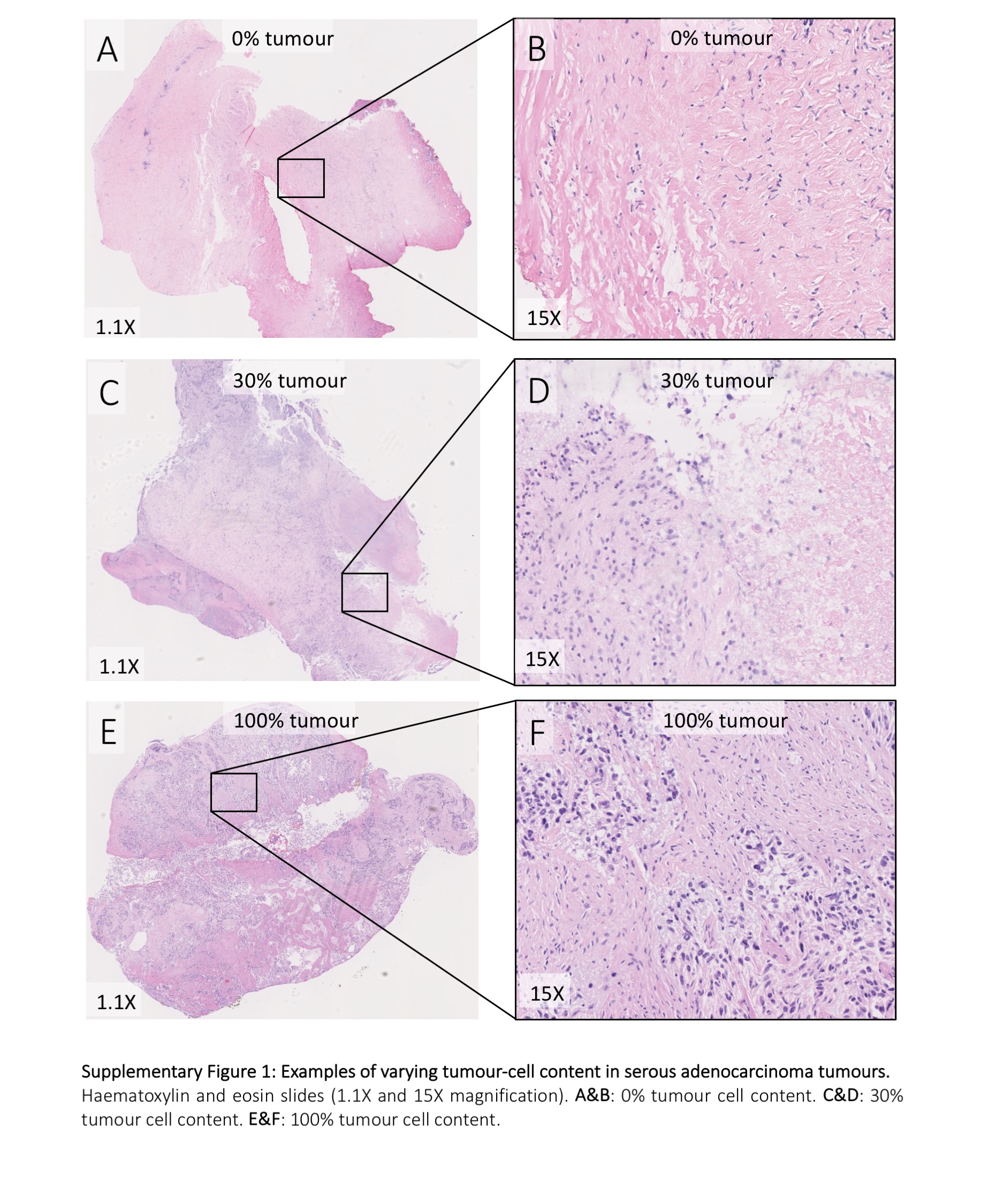

Supplement: Supplementary file 1 — Supplementary Figure 1: Examples of varying tumour-cell content in serous adenocarcinoma tumours [file 41416_2018_48_MOESM1_ESM.jpg]
