# Supplementary material for: The surgical intelligent knife distinguishes normal, borderline and malignant gynaecological tissues using rapid evaporative ionisation mass spectrometry (REIMS)
Source: Br J Cancer. 2018 Apr 19;118(10):1349–58. doi: 10.1038/s41416-018-0048-3 (PMC5959892; doi:10.1038/s41416-018-0048-3)
Supplement: Supplementary file 2 — Supplementary Table 1: Characteristics of all frozen tumour samples [file 41416_2018_48_MOESM2_ESM.docx]

|  | **OC** | **BOT** | **Benign** |  |
| --- | --- | --- | --- | --- |
|  | n=63 | n=21 | n=22 |  |
| **Histology** |  |  |  |  |
| High Grade Serous | 36 | 7 | 6 |  |
| Endometrioid | 11 | 0 | 1 |  |
| Clear cell | 9 | 0 | 0 |  |
| Mucinous | 7 | 11 | 0 |  |
| Mixed | 0 | 2 | 0 |  |
| Other | 0 | 0 | 3 |  |
| Fibroma NOS | NA | NA | 5 |  |
| dna | 0 | 1 | 7 |  |
| **Grade** |  |  |  |  |
| 1 | 3 | NA | NA |  |
| 2 | 10 | NA | NA |  |
| 3 | 47 | NA | NA |  |
| dna | 3 | NA | NA |  |
| **Stage** |  |  |  |  |
| I | 8 | 12 | NA |  |
| II | 7 | 0 | NA |  |
| III | 31 | 0 | NA |  |
| IV | 6 | 0 | NA |  |
| dna | 11 | 9 | NA |  |
|  |  |  |  |  |
| dna: data not available, NA: not applicable, NOS: not otherwise specified | | | | |

#### Supplementary Table 1:

#### Characteristics of all frozen tumour samples

All cancer, borderline and benign frozen samples included in the study and their tumour characteristics. Normal samples; ovary (n=15), fallopian tube (n=15), peritoneum (n=14) not shown.
