# Supplementary material for: The surgical intelligent knife distinguishes normal, borderline and malignant gynaecological tissues using rapid evaporative ionisation mass spectrometry (REIMS)
Source: Br J Cancer. 2018 Apr 19;118(10):1349–58. doi: 10.1038/s41416-018-0048-3 (PMC5959892; doi:10.1038/s41416-018-0048-3)
Supplement: Supplementary file 3 — Supplementary table 2: Fresh samples collected and processed with the iKnife. Patients n=35, samples n=119 [file 41416_2018_48_MOESM3_ESM.docx]

**Supplementary table 2:**

**Fresh samples collected and processed with the iKnife. Patients n=35, samples n=119**

OC = ovarian cancer, BOT = borderline ovarian tumour, HGSOC = high grade serous OC

| **ID** | **Tissue** | **Histology** |
| --- | --- | --- |
| OC_001_A | HGSOC | Serous OC |
| OC_001_B | Peritoneum | Normal |
| OC_001_C | Fallopian | Normal |
| OC_001_D | Omentum | Normal |
| OC_002_A | Omentum | Normal |
| OC_002_B | Peritoneum | Normal |
| OC_002_C | Fallopian | Normal |
| OC_002_D | Ovary | Endometrioid endometrial |
| OC_003_A | Peritoneum | Normal |
| OC_003_B | Ovary | Normal |
| OC_003_C | Tumour | Cellular leiomyoma |
| OC_003_D | Tumour | Cellular leiomyoma |
| OC_003_E | Fallopian | Normal |
| OC_004_A | Ovary | Normal |
| OC_004_B | Fallopian | Normal |
| OC_005_A | HGSOC | Mixed OC |
| OC_005_B | Fallopian | Normal |
| OC_005_C | Omentum | Normal |
| OC_007_A | Fallopian | Serous OC |
| OC_007_B | Omentum | Serous OC |
| OC_007_C | Peritoneum | Serous OC |
| OC_007_D | Peritoneum | Serous OC |
| OC_007_E | HGSOC | Serous OC |
| OC_008_A | Ovary | Normal |
| OC_008_B | Fallopian | Normal |
| OC_008_C | Omentum | Normal |
| OC_009_A | Ovary | Normal |
| OC_014_A | Omentum | Serous OC |
| OC_014_B | Omentum | Normal |
| OC_014_C | HGSOC | Serous OC |
| OC_014_D | HGSOC | Serous OC |
| OC_015_A | Omentum | Normal |
| OC_016_A | Peritoneum | Nodule: Granulosa |
| OC_017_A | Omentum | Serous OC |
| OC_017_B | Peritoneum | Serous OC |
| OC_017_C | Peritoneum | Serous OC |
| OC_018_A | Ovary | Normal |
| OC_018_B | Fallopian | Normal |
| OC_018_C | Peritoneum | Normal |
| OC_019_A | Fallopian | Normal |
| OC_019_B | Ovary | Normal |
| OC_019_C | Peritoneum | Normal |
| OC_019_D | Tumour | Endometrial carcinoma |
| OC_020_A | Omentum | Mucinous |
| OC_020_B | Peritoneum | Mucinous |
| OC_020_C | Ovary | Normal |
| OC_020_D | HGSOC | Mucinous |
| OC_021_A | HGSOC | Serous OC |
| OC_021_B | Fallopian | Normal |
| OC_021_C | Peritoneum | Normal |
| OC_022_A | OC | Carcinosarcoma |
| OC_022_B | OC | Carcinosarcoma |
| OC_022_C | OC | Carcinosarcoma |
| OC_022_D | Peritoneum | Carcinosarcoma |
| OC_022_E | Omentum | Carcinosarcoma |
| OC_025_A | HGSOC | Serous OC |
| OC_025_B | Omentum | Normal |
| OC_025_C | Omentum | Serous OC |
| OC_025_D | Peritoneum | Normal |
| OC_025_E | Peritoneum | Serous OC |
| OC_025_F | Fat | Normal |
| OC_026_A | HGSOC | Serous OC |
| OC_026_B | Omentum | Serous OC |
| OC_026_C | Peritoneum | Serous OC |
| OC_027_A | HGSOC | Serous OC |
| OC_027_B | Omentum | Normal |
| OC_027_C | Omentum | Serous OC |
| OC_028_A | HGSOC | Serous OC |
| OC_028_B | Peritoneum | Normal |
| OC_028_C | Omentum | Normal |
| OC_028_D | Omentum | Serous OC |
| OC_029_A | Fallopian | Normal |
| OC_029_B | HGSOC | Carcinoma |
| OC_029_C | Omentum | Carcinoma |
| OC_029_D | Omentum | Normal |
| OC_030_A | Ovary | Benign cystadenofibroma |
| OC_030_B | Omentum | Normal |
| OC_030_C | Fallopian | Normal |
| OC_030_D | Myometrium | Normal |
| OC_031_A | OC | Carcinosarcoma |
| OC_031_B | Omentum | Normal |
| OC_031_C | Myometrium | Normal |
| OC_032_A | OC | Carcinoma |
| OC_032_B | Omentum | Carcinoma |
| OC_032_C | Peritoneum | Carcinoma |
| OC_032_D | Peritoneum | Normal |
| OC_033_A | Ovary | Normal |
| OC_033_B | Fallopian | Normal |
| OC_034_A | HGSOC | Serous OC |
| OC_034_B | Omentum | Serous OC |
| OC_034_C | Peritoneum | Serous OC |
| OC_034_D | Fallopian | Normal |
| OC_035_A | OC | Carcinoma |
| OC_035_B | Peritoneum | Carcinoma |
| OC_036_A | Omentum | Serous OC |
| OC_037_A | Ovary | Normal |
| OC_037_B | Fallopian | Normal |
| OC_038_A | Ovary | Normal |
| OC_038_B | Fallopian | Normal |
| OC_038_C | Tumour | Endometrial carcinoma |
| OC_039_A | Ovary | Normal |
| OC_039_B | Fallopian | Normal |
| OC_039_C | Fibroid | Leiomyoma |
| OC_039_D | Fibroid | Leiomyoma |
| OC_039_E | Omentum | Normal |
| OC_039_F | Peritoneum | Normal |
| OC_040_A | HGSOC | Serous OC |
| OC_040_B | Omentum | Serous OC |
| OC_040_C | Omentum | Normal |
| OC_040_D | Ovary | Normal |
| OC_040_E | Fallopian | Normal |
| OC_040_F | Omentum | Normal |
| OC_041_A | Ovary | Normal |
| OC_041_B | Fallopian | Normal |
| OC_042_A | BOT | BOT |
| OC_042_B | Omentum | BOT |
| OC_042_C | Peritoneum | BOT |
| OC_042_D | Peritoneum | BOT |
| OC_042_E | Peritoneum | BOT |
|  |  |  |
|  |  |  |
