# Supplementary material for: The surgical intelligent knife distinguishes normal, borderline and malignant gynaecological tissues using rapid evaporative ionisation mass spectrometry (REIMS)
Source: Br J Cancer. 2018 Apr 19;118(10):1349–58. doi: 10.1038/s41416-018-0048-3 (PMC5959892; doi:10.1038/s41416-018-0048-3)
Supplement: Supplementary file 4 — Supplementary Table 3: Leave one patient out cross-validated classification of ovarian cancer and normal tissues [file 41416_2018_48_MOESM4_ESM.docx]

#### Supplementary Table 3:

#### Leave one patient out cross-validated classification of ovarian cancer and normal tissues.

The cross validation shows the accuracy of correct classification for each of the tissue classes in the model shown in Figure 2. Overall correct classification of all spectra 97.6%.

| **Overall correct classification 97·6%** | | **Predicted class** | | | |
| --- | --- | --- | --- | --- | --- |
|  |  | Normal fallopian | Normal ovary | Normal peritoneum | Ovarian cancer |
| **Actual class** | Normal fallopian | 100% | 0% | 0% | 0% |
|  | Normal  ovary | 6·7% | 93·3% | 0% | 0% |
|  | Normal peritoneum | 0% | 0% | 100% | 0% |
|  | Ovarian  cancer | 0% | 0% | 2·6% | 97·4% |
