# Supplementary material for: The surgical intelligent knife distinguishes normal, borderline and malignant gynaecological tissues using rapid evaporative ionisation mass spectrometry (REIMS)
Source: Br J Cancer. 2018 Apr 19;118(10):1349–58. doi: 10.1038/s41416-018-0048-3 (PMC5959892; doi:10.1038/s41416-018-0048-3)
Supplement: Supplementary file 5 — Supplementary Table 4: [file 41416_2018_48_MOESM5_ESM.docx]

| **Tissue Class** | **Samples** | **Burns** | **>75% probability** | **Correct report** | **Diagnostic accuracy (%)** |
| --- | --- | --- | --- | --- | --- |
| Ovarian cancer | 7 | 65 | 61 | 61 | 100.0 |
| Normal ovary | 9 | 69 | 64 | 64 | 100.0 |
| Fallopian tube | 11 | 61 | 58 | 58 | 100.0 |
| Peritoneum | 5 | 37 | 34 | 32 | 94.1 |
| **TOTALS** | **32** | **232** | **217** | **215** |  |

#### Supplementary Table 4:

#### Fresh tissue samples classified using frozen cut model as reference dataset (cut mode)
