# Supplementary material for: The surgical intelligent knife distinguishes normal, borderline and malignant gynaecological tissues using rapid evaporative ionisation mass spectrometry (REIMS)
Source: Br J Cancer. 2018 Apr 19;118(10):1349–58. doi: 10.1038/s41416-018-0048-3 (PMC5959892; doi:10.1038/s41416-018-0048-3)
Supplement: Supplementary file 6 — Supplementary Table 5: [file 41416_2018_48_MOESM6_ESM.docx]

**Supplementary Table 5:**

**Multiple raters’ impressions of tissue type (Surgeon vs Histopathologist vs iKnife)**

|  | **SAMPLE INFORMATION** | | | **SURGEON'S IMPRESSION** | | | **HISTOPATHOLOGISTS IMPRESSION** | | | | **IKNIFE IMPRESSION** | |
| --- | --- | --- | --- | --- | --- | --- | --- | --- | --- | --- | --- | --- |
| **Burn** | **Burn ID** | **Diathemy mode** | **Tissue** | **Description** | **Class** | **Comments** | **Histological diagnosis** | **Class** | **Tumour content (%)** | **Distance from tumour (mm)** | **Class** | **% probability** |
| 1 | OC_007_C_CG_1 | Coagulation | Peritoneum | nodule | Cancer | came from diaphragm | Serous carcinoma of ovary | Cancer | 100 |  | Normal | 90.1% |
| 2 | OC_007_C_CG_2 | Coagulation | Peritoneum | nodule | Cancer | came from diaphragm | Serous carcinoma of ovary | Cancer | 100 |  | Cancer | 98.0% |
| 3 | OC_007_C_CG_3 | Coagulation | Peritoneum | nodule | Cancer | came from diaphragm | Serous carcinoma of ovary | Cancer | 100 |  | Cancer | 72.8% |
| 4 | OC_007_C_CG_4 | Coagulation | Peritoneum | nodule | Cancer | came from diaphragm | Serous carcinoma of ovary | Cancer | 50 |  | Cancer | 98.4% |
| 5 | OC_007_C_CG_5 | Coagulation | Peritoneum | nodule | Cancer | came from diaphragm | Serous carcinoma of ovary | Cancer | 90 |  | Cancer | 98.5% |
| 6 | OC_007_C_CG_6 | Coagulation | Peritoneum | nodule | Cancer | came from diaphragm | Serous carcinoma of ovary | Cancer | 50 |  | Normal | 92.2% |
| 7 | OC_007_C_CG_7 | Coagulation | Peritoneum | ?normal | Normal | came from diaphragm | muscle + adipose tissue | Normal |  | 1.7 | Normal | 95.2% |
| 8 | OC_007_C_CG_8 | Coagulation | Peritoneum | ?normal | Normal | came from diaphragm | muscle + adipose tissue | Normal |  | 3.5 | Normal | 98.0% |
| 9 | OC_007_C_CG_9 | Coagulation | Peritoneum | ?normal | Normal | came from diaphragm | muscle + adipose tissue + lymphocytes | Normal |  | 1.8 | Normal | 98.1% |
| 10 | OC_007_C_CG_10 | Coagulation | Peritoneum | ?normal | Normal | came from diaphragm | muscle + adipose tissue | Normal |  | 1.1 | Normal | 98.7% |
| 11 | OC_007_C_CG_11 | Coagulation | Peritoneum | ?normal | Normal | came from diaphragm | muscle + adipose tissue | Normal |  | 2.6 | Normal | 99.0% |
| 12 | OC_007_C_CG_12 | Coagulation | Peritoneum | ?normal | Normal | came from diaphragm | muscle + adipose tissue | Normal |  | 0.7 | Normal | 98.0% |
| 13 | OC_007_C_CT_1 | Cut | Peritoneum | nodule | Cancer | came from diaphragm | Serous carcinoma of ovary | Cancer | 100 |  | Cancer | 82.9% |
| 14 | OC_007_C_CT_2 | Cut | Peritoneum | nodule | Cancer | came from diaphragm | Serous carcinoma of ovary | Cancer | 100 |  | Cancer | 98.3% |
| 15 | OC_007_C_CT_3 | Cut | Peritoneum | nodule edge | Normal | came from diaphragm | Fibrous tissue | Normal |  | 0.1 | Normal | 52.8% |
| 16 | OC_007_C_CT_4 | Cut | Peritoneum | nodule edge | Normal | came from diaphragm | Fibrous tissue | Normal |  | 0.6 | Normal | 90.9% |
| 17 | OC_007_C_CT_5 | Cut | Peritoneum | ?normal | Normal | came from diaphragm | Fibrous tissue + adipose tissue | Normal |  | 2.1 | Normal | 91.1% |
| 18 | OC_007_C_CT_6 | Cut | Peritoneum | nodule edge | Normal | came from diaphragm | Fibrous tissue + adipose tissue + lymphocytes | Normal |  | 0.4 | Normal | 92.7% |
| 19 | OC_007_C_CT_7 | Cut | Peritoneum | near tumour | Normal | came from diaphragm | Fibrous tissue + adipose tissue | Normal |  | 1.4 | Normal | 92.0% |
| 20 | OC_007_C_CT_8 | Cut | Peritoneum | near tumour | Normal | came from diaphragm | Fibrous tissue + adipose tissue | Normal |  | 0.9 | Normal | 86.9% |
| 21 | OC_007_C_CT_9 | Cut | Peritoneum | >1cm from tumour | Normal | came from diaphragm | Fibrous tissue | Normal |  | 0.3 | Outlier | 0.0% |
| 22 | OC_007_C_CT_10 | Cut | Peritoneum | >1cm from tumour | Normal | came from diaphragm | Fibrous tissue + adipose tissue | Normal |  | 0.6 | Normal | 88.6% |
| 23 | OC_007_C_CT_11 | Cut | Peritoneum | ?normal | Normal | came from diaphragm | Fibrous tissue + adipose tissue | Normal |  | 3.4 | Normal | 92.9% |
| 24 | OC_007_C_CT_12 | Cut | Peritoneum | ?normal | Normal | came from diaphragm | Fibrous tissue + adipose tissue + lymphocytes | Normal |  | 4.2 | Normal | 90.2% |
| 25 | OC_007_C_CT_13 | Cut | Peritoneum | near tumour | Normal | came from diaphragm | Serous carcinoma of ovary | Cancer | 60 |  | Cancer | 92.4% |
| 26 | OC_007_C_CT_14 | Cut | Peritoneum | near tumour | Normal | came from diaphragm | Fibrous tissue + adipose tissue | Normal |  | 0.9 | Cancer | 93.3% |
| 27 | OC_007_C_CT_15 | Cut | Peritoneum | near tumour | Normal | came from diaphragm | Serous carcinoma of ovary | Cancer | 10 |  | Cancer | 93.5% |
| 28 | OC_007_C_CT_16 | Cut | Peritoneum | ?normal | Normal | came from diaphragm | Fibrous tissue + adipose tissue | Normal |  | 0.7 | Normal | 95.7% |
| 29 | OC_007_D_CG_1 | Coagulation | Peritoneum | nodule | Cancer |  | Serous carcinoma of ovary | Cancer | 100 |  | Cancer | 96.8% |
| 30 | OC_007_D_CG_2 | Coagulation | Peritoneum | nodule | Cancer |  | Serous carcinoma of ovary | Cancer | 100 |  | Cancer | 98.6% |
| 31 | OC_007_D_CG_3 | Coagulation | Peritoneum | nodule | Cancer |  | Serous carcinoma of ovary | Cancer | 90 |  | Cancer | 97.6% |
| 32 | OC_007_D_CG_4 | Coagulation | Peritoneum | nodule | Cancer |  | Serous carcinoma of ovary | Cancer | 15 |  | Cancer | 96.1% |
| 33 | OC_007_D_CG_5 | Coagulation | Peritoneum | ?normal | Normal |  | Fibrous tissue + lymphocytes | Normal |  | 1.8 | Cancer | 96.0% |
| 34 | OC_007_D_CG_6 | Coagulation | Peritoneum | ?normal | Normal |  | Serous carcinoma of ovary | Cancer | 30 |  | Cancer | 78.2% |
| 35 | OC_007_D_CT_1 | Cut | Peritoneum | nodule | Cancer |  | Serous carcinoma of ovary | Cancer | 80 |  | Cancer | 96.1% |
| 36 | OC_007_D_CT_2 | Cut | Peritoneum | nodule | Cancer |  | Serous carcinoma of ovary | Cancer | 90 |  | Cancer | 97.6% |
| 37 | OC_007_D_CT_3 | Cut | Peritoneum | nodule | Cancer |  | Serous carcinoma of ovary | Cancer | 100 |  | Cancer | 97.4% |
| 38 | OC_007_D_CT_4 | Cut | Peritoneum | nodule | Cancer |  | Serous carcinoma of ovary | Cancer | 100 |  | Cancer | 93.2% |
| 39 | OC_007_D_CT_5 | Cut | Peritoneum | nodule | Cancer |  | Serous carcinoma of ovary | Cancer | 90 |  | Cancer | 93.4% |
| 40 | OC_007_D_CT_6 | Cut | Peritoneum | nodule | Cancer |  | Serous carcinoma of ovary | Cancer | 100 |  | Cancer | 98.0% |
| 41 | OC_007_D_CT_7 | Cut | Peritoneum | nodule | Cancer |  | Serous carcinoma of ovary | Cancer | 100 |  | Cancer | 86.3% |
| 42 | OC_007_D_CT_8 | Cut | Peritoneum | nodule | Cancer |  | Serous carcinoma of ovary | Cancer | 100 |  | Cancer | 97.5% |
|  | **SAMPLE INFORMATION** | | | **SURGEON'S IMPRESSION** | | | **HISTOPATHOLOGISTS IMPRESSION** | | | | **IKNIFE IMPRESSION** | |
| **Burn** | **Burn ID** | **Diathemy mode** | **Tissue** | **Description** | **Class** | **Comments** | **Histological diagnosis** | **Class** | **Tumour content (%)** | **Distance from tumour (mm)** | **Class** | **% probability** |
| 43 | OC_007_D_CT_9 | Cut | Peritoneum | nodule | Cancer |  | Serous carcinoma of ovary | Cancer | 100 |  | Cancer | 98.8% |
| 44 | OC_007_D_CT_10 | Cut | Peritoneum | nodule | Cancer |  | Serous carcinoma of ovary | Cancer | 90 |  | Cancer | 98.8% |
| 45 | OC_007_D_CT_11 | Cut | Peritoneum | ?normal | Normal | close to tumour | Fibrous tissue + lymphocytes | Normal | 0 | 0.1 | Cancer | 84.6% |
| 46 | OC_007_D_CT_12 | Cut | Peritoneum | ?normal | Normal | close to tumour | Serous carcinoma of ovary | Cancer | 40 |  | Cancer | 93.3% |
| 47 | OC_007_D_CT_13 | Cut | Peritoneum | ?normal | Normal | close to tumour | Fibrous tissue + lymphocytes | Normal |  | 0.4 | Cancer | 90.4% |
| 48 | OC_007_D_CT_14 | Cut | Peritoneum | ?normal | Normal | close to tumour | Fibrous tissue + lymphocytes | Normal |  | 0.2 | Normal | 66.1% |
| 49 | OC_017_A_CG_1 | Coagulation | Omentum | ?all tumour | Cancer | generally infiltrated | Burn not seen but generally infiltrated | Cancer |  |  | Normal | 97.6% |
| 50 | OC_017_A_CG_2 | Coagulation | Omentum | ?all tumour | Cancer | generally infiltrated | Serous carcinoma of ovary | Cancer | 50 |  | Normal | 92.9% |
| 51 | OC_017_A_CG_3 | Coagulation | Omentum | ?all tumour | Cancer | generally infiltrated | Serous carcinoma of ovary | Cancer | 30 |  | Normal | 93.6% |
| 52 | OC_017_A_CG_4 | Coagulation | Omentum | ?all tumour | Cancer | generally infiltrated | Serous carcinoma of ovary | Cancer | 5 |  | Normal | 97.7% |
| 53 | OC_017_A_CG_5 | Coagulation | Omentum | ?all tumour | Cancer | generally infiltrated | Serous carcinoma of ovary | Cancer | 70 |  | Normal | 94.5% |
| 54 | OC_017_A_CG_6 | Coagulation | Omentum | ?all tumour | Cancer | generally infiltrated | Serous carcinoma of ovary | Cancer | 50 |  | Normal | 84.5% |
| 55 | OC_017_A_CG_7 | Coagulation | Omentum | ?all tumour | Cancer | generally infiltrated | Serous carcinoma of ovary | Cancer | 60 |  | Normal | 90.7% |
| 56 | OC_017_A_CG_8 | Coagulation | Omentum | ?all tumour | Cancer | generally infiltrated | Serous carcinoma of ovary | Cancer | 40 |  | Normal | 91.2% |
| 57 | OC_017_A_CG_9 | Coagulation | Omentum | ?all tumour | Cancer | generally infiltrated | Serous carcinoma of ovary | Cancer | 50 |  | Normal | 96.9% |
| 58 | OC_017_A_CT_1 | Cut | Omentum | ?all tumour | Cancer | generally infiltrated | Serous carcinoma of ovary | Cancer | 60 |  | Cancer | 98.8% |
| 59 | OC_017_A_CT_2 | Cut | Omentum | ?all tumour | Cancer | generally infiltrated | Serous carcinoma of ovary | Cancer | 80 |  | Cancer | 99.0% |
| 60 | OC_017_A_CT_3 | Cut | Omentum | ?all tumour | Cancer | generally infiltrated | Serous carcinoma of ovary | Cancer | 5 |  | Cancer | 98.8% |
| 61 | OC_017_A_CT_4 | Cut | Omentum | ?all tumour | Cancer | generally infiltrated | Serous carcinoma of ovary | Cancer | 60 |  | Cancer | 98.8% |
| 62 | OC_017_A_CT_5 | Cut | Omentum | ?all tumour | Cancer | generally infiltrated | Serous carcinoma of ovary | Cancer | 50 |  | Cancer | 98.8% |
| 63 | OC_017_A_CT_6 | Cut | Omentum | ?all tumour | Cancer | generally infiltrated | Serous carcinoma of ovary | Cancer | 50 |  | Cancer | 98.7% |
| 64 | OC_017_A_CT_7 | Cut | Omentum | ?all tumour | Cancer | generally infiltrated | Serous carcinoma of ovary | Cancer | 60 |  | Cancer | 98.8% |
| 65 | OC_017_A_CT_8 | Cut | Omentum | ?all tumour | Cancer | generally infiltrated | Serous carcinoma of ovary | Cancer | 80 |  | Cancer | 98.8% |
| 66 | OC_017_A_CT_9 | Cut | Omentum | ?all tumour | Cancer | generally infiltrated | Serous carcinoma + Fibrous and adipose tissue | Cancer | 2 |  | Cancer | 95.6% |
| 67 | OC_020_A_CG_a | Coagulation | Omentum | nodule | Cancer |  | Mucinous carcinoma of ovary | Cancer | up to 50 |  | Cancer | 91.3% |
| 68 | OC_020_A_CG_b | Coagulation | Omentum | nodule | Cancer |  | Mucinous carcinoma of ovary | Cancer | up to 50 |  | Cancer | 94.4% |
| 69 | OC_020_A_CG_c | Coagulation | Omentum | nodule | Cancer |  | Mucinous carcinoma of ovary | Cancer | up to 50 |  | Cancer | 92.9% |
| 70 | OC_020_A_CG_d | Coagulation | Omentum | nodule | Cancer |  | Fibrous tissue + adipose tissue | Normal |  | 1.2 | Cancer | 91.9% |
| 71 | OC_020_A_CG_e | Coagulation | Omentum | nodule | Cancer |  | Fibrous tissue + adipose tissue | Normal |  |  | Cancer | 97.9% |
| 72 | OC_020_A_CG_f | Coagulation | Omentum | nodule | Cancer |  | Fibrous tissue + adipose tissue | Normal |  |  | Normal | 74.2% |
| 73 | OC_020_A_CG_g | Coagulation | Omentum | ?normal | Normal |  | Fibrous tissue + adipose tissue | Normal |  | 5.5 | Normal | 93.6% |
| 74 | OC_020_A_CG_h | Coagulation | Omentum | ?normal | Normal |  | Fibrous tissue + adipose tissue | Normal |  |  | Normal | 96.3% |
| 75 | OC_020_A_CG_i | Coagulation | Omentum | ?normal | Normal |  | Fibrous tissue + adipose tissue | Normal |  |  | Normal | 96.9% |
| 76 | OC_020_A_CT_1 | Cut | Omentum | nodule | Cancer |  | Mucinous carcinoma of ovary | Cancer | 80 |  | Cancer | 90.7% |
| 77 | OC_020_A_CT_2 | Cut | Omentum | nodule | Cancer |  | Mucinous carcinoma of ovary | Cancer | 40 |  | Cancer | 91.4% |
| 78 | OC_020_A_CT_3 | Cut | Omentum | nodule | Cancer |  | Mucinous carcinoma of ovary | Cancer | 40 |  | Cancer | 80.4% |
| 79 | OC_020_A_CT_4 | Cut | Omentum | superficial nodule | Cancer |  | Mucinous carcinoma of ovary | Cancer | 80 |  | Cancer | 92.2% |
| 80 | OC_020_A_CT_5 | Cut | Omentum | superficial nodule | Cancer |  | Mucinous carcinoma of ovary | Cancer | 90 |  | Cancer | 82.8% |
| 81 | OC_020_A_CT_6 | Cut | Omentum | superficial nodule | Cancer |  | Mucinous carcinoma of ovary | Cancer | 80 |  | Cancer | 88.6% |
| 82 | OC_020_A_CT_7 | Cut | Omentum | nodule | Cancer |  | Mucinous carcinoma of ovary | Cancer | 40 |  | Cancer | 69.3% |
| 83 | OC_020_A_CT_8 | Cut | Omentum | nodule | Cancer |  | Mucinous carcinoma of ovary | Cancer | 30 |  | Cancer | 84.7% |
| 84 | OC_020_A_CT_9 | Cut | Omentum | close to nodule | Normal | 0.5cm from edge nodule | Mucinous carcinoma of ovary | Cancer | 60 |  | Normal | 96.0% |
|  |  |  |  |  |  |  |  |  |  |  |  |  |
|  | **SAMPLE INFORMATION** | | | **SURGEON'S IMPRESSION** | | | **HISTOPATHOLOGISTS IMPRESSION** | | | | **IKNIFE IMPRESSION** | |
| **Burn** | **Burn ID** | **Diathemy mode** | **Tissue** | **Description** | **Class** | **Comments** | **Histological diagnosis** | **Class** | **Tumour content (%)** | **Distance from tumour (mm)** | **Class** | **% probability** |
| 85 | OC_020_A_CT_10 | Cut | Omentum | close to nodule | Normal | 0.5cm from edge nodule | Mucinous carcinoma of ovary | Cancer | 25 |  | Normal | 97.8% |
| 86 | OC_020_A_CT_11 | Cut | Omentum | close to nodule | Normal | 0.5cm from edge nodule | Mucinous carcinoma of ovary | Cancer | 25 |  | Normal | 84.6% |
| 87 | OC_020_A_CT_12 | Cut | Omentum | close to nodule | Normal | 1cm from edge nodule | Fibrous tissue + adipose tissue + mesothelium | Normal |  | 5.1 | Normal | 91.1% |
| 88 | OC_020_A_CT_13 | Cut | Omentum | close to nodule | Normal | 1cm from edge nodule | Fibrous tissue + adipose tissue | Normal |  | 3.0 | Normal | 85.4% |
| 89 | OC_020_A_CT_14 | Cut | Omentum | close to nodule | Normal | 1cm from edge nodule | Fibrous tissue + adipose tissue | Normal |  | 3.8 | Normal | 62.3% |
| 90 | OC_020_B_CG_1 | Coagulation | Peritoneum | nodule | Cancer |  | Fibrous tissue + adipose tissue | Normal |  | 4.9 | Normal | 93.4% |
| 91 | OC_020_B_CG_2 | Coagulation | Peritoneum | nodule | Cancer |  | Fibrous tissue + adipose tissue | Normal |  | 3.4 | Normal | 89.6% |
| 92 | OC_020_B_CG_3 | Coagulation | Peritoneum | nodule | Cancer |  | Fibrous tissue + adipose tissue + lymphocytes | Normal |  | 4.5 | Normal | 96.7% |
| 93 | OC_020_B_CG_4 | Coagulation | Peritoneum | nodule | Cancer |  | Mucinous carcinoma of ovary | Cancer | 60 |  | Normal | 94.6% |
| 94 | OC_020_B_CG_5 | Coagulation | Peritoneum | nodule | Cancer |  | Fibrous tissue + lymphocytes | Normal |  | 3.6 | Normal | 90.3% |
| 95 | OC_020_B_CG_6 | Coagulation | Peritoneum | nodule | Cancer |  | Fibrous tissue + acute inflammation | Normal |  | 0.6 | Normal | 90.5% |
| 96 | OC_020_B_CG_7 | Coagulation | Peritoneum | ?normal | Normal |  | Fibrous tissue + lymphocytes | Normal |  | 3.3 | Normal | 96.0% |
| 97 | OC_020_B_CG_8 | Coagulation | Peritoneum | ?normal | Normal |  | Mucinous carcinoma of ovary | Cancer | 20 |  | Normal | 91.4% |
| 98 | OC_020_B_CG_9 | Coagulation | Peritoneum | ?normal | Normal |  | Fibrous tissue + acute inflammation | Normal |  | 0.5 | Normal | 86.9% |
| 99 | OC_020_B_CG_10 | Coagulation | Peritoneum | ?normal | Normal |  | Mucinous carcinoma of ovary | Cancer | 50 |  | Normal | 97.5% |
| 100 | OC_020_B_CT_1 | Cut | Peritoneum | nodule | Cancer |  | Mucinous carcinoma of ovary | Cancer | 15 |  | Cancer | 61.8% |
| 101 | OC_020_B_CT_2 | Cut | Peritoneum | nodule | Cancer |  | Mucinous carcinoma of ovary | Cancer | 40 |  | Normal | 76.9% |
| 102 | OC_020_B_CT_3 | Cut | Peritoneum | nodule | Cancer |  | Fibrous tissue + adipose tissue + lymphocytes | Normal |  | 0.8 | Cancer | 76.2% |
| 103 | OC_020_B_CT_4 | Cut | Peritoneum | nodule | Cancer |  | Mucinous carcinoma of ovary | Cancer | 60 |  | Cancer | 69.8% |
| 104 | OC_020_B_CT_5 | Cut | Peritoneum | nodule | Cancer |  | Mucinous carcinoma of ovary | Cancer | 90 |  | Cancer | 70.6% |
| 105 | OC_020_B_CT_6 | Cut | Peritoneum | nodule | Cancer |  | Mucinous carcinoma of ovary | Cancer | 90 |  | Cancer | 74.6% |
| 106 | OC_020_B_CT_7 | Cut | Peritoneum | nodule | Cancer |  | Mucinous carcinoma of ovary | Cancer | 10 |  | Cancer | 78.1% |
| 107 | OC_020_B_CT_8 | Cut | Peritoneum | nodule | Cancer |  | Mucinous carcinoma of ovary | Cancer | 30 |  | Cancer | 74.7% |
| 108 | OC_020_B_CT_9 | Cut | Peritoneum | nodule | Cancer |  | Mucinous carcinoma of ovary | Cancer | 30 |  | Cancer | 73.0% |
| 109 | OC_020_B_CT_10 | Cut | Peritoneum | close to nodule | Normal | tumour edge | Fibrous tissue + lymphocytes | Normal |  | 0.5 | Normal | 91.5% |
| 110 | OC_020_B_CT_11 | Cut | Peritoneum | close to nodule | Normal | ~3mm from edge | Fibrous tissue + lymphocytes | Normal |  | 0.5 | Normal | 74.9% |
| 111 | OC_020_B_CT_12 | Cut | Peritoneum | ?normal | Normal | ~6mm from edge | Fibrous tissue + adipose tissue + lymphocytes | Normal |  | 1.7 | Normal | 88.8% |
| 112 | OC_020_B_CT_13 | Cut | Peritoneum | close to nodule (7,8,9) | Normal | ~2mm from 7,8,9 nodule | Fibrous tissue + adipose tissue + lymphocytes | Normal |  | 0.9 | Normal | 87.2% |
| 113 | OC_020_B_CT_14 | Cut | Peritoneum | ?normal | Normal | ~3mm from 7,8,9 nodule | Mucinous carcinoma of ovary | Cancer | 50 |  | Normal | 85.1% |
| 114 | OC_020_B_CT_15 | Cut | Peritoneum | ?normal | Normal | ~6mm from 7,8,9 nodule | Fibrous tissue + adipose tissue | Normal |  | 1.6 | Normal | 92.9% |
| 115 | OC_022_D_CG_1 | Coagulation | Peritoneum | nodule | Cancer |  | Carcinosarcoma | Cancer | 100 |  | Cancer | 90.3% |
| 116 | OC_022_D_CG_2 | Coagulation | Peritoneum | nodule | Cancer |  | Carcinosarcoma | Cancer | 100 |  | Cancer | 99.6% |
| 117 | OC_022_D_CG_3 | Coagulation | Peritoneum | nodule | Cancer |  | Carcinosarcoma | Cancer | 100 |  | Cancer | 97.8% |
| 118 | OC_022_D_CG_4 | Coagulation | Peritoneum | nodule | Cancer |  | Carcinosarcoma | Cancer | 100 |  | Cancer | 95.3% |
| 119 | OC_022_D_CG_5 | Coagulation | Peritoneum | ?normal | Normal | close to tumour | Fibrous tissue + lymphocytes | Normal |  | 3.4 | Normal | 97.5% |
| 120 | OC_022_D_CG_6 | Coagulation | Peritoneum | ?normal | Normal | close to tumour | Fibrous tissue + lymphocytes | Normal |  |  | Normal | 97.8% |
| 121 | OC_022_D_CG_7 | Coagulation | Peritoneum | ?normal | Normal | far from nodule | Fibrous tissue + lymphocytes | Normal |  | 10.1 | Normal | 98.4% |
| 122 | OC_022_D_CG_8 | Coagulation | Peritoneum | ?normal | Normal | far from nodule | Fibrous tissue + lymphocytes | Normal |  |  | Normal | 84.2% |
| 123 | OC_022_D_CT_1 | Cut | Peritoneum | nodule | Cancer |  | Carcinosarcoma | Cancer | 100 |  | Cancer | 98.5% |
| 124 | OC_022_D_CT_2 | Cut | Peritoneum | nodule | Cancer |  | Carcinosarcoma | Cancer | 100 |  | Cancer | 98.8% |
| 125 | OC_022_D_CT_3 | Cut | Peritoneum | nodule | Cancer |  | Carcinosarcoma | Cancer | 100 |  | Cancer | 98.7% |
| 126 | OC_022_D_CT_4 | Cut | Peritoneum | nodule | Cancer |  | Carcinosarcoma | Cancer | 100 |  | Cancer | 98.8% |
|  |  |  |  |  |  |  |  |  |  |  |  |  |
|  | **SAMPLE INFORMATION** | | | **SURGEON'S IMPRESSION** | | | **HISTOPATHOLOGISTS IMPRESSION** | | | | **IKNIFE IMPRESSION** | |
| **Burn** | **Burn ID** | **Diathemy mode** | **Tissue** | **Description** | **Class** | **Comments** | **Histological diagnosis** | **Class** | **Tumour content (%)** | **Distance from tumour (mm)** | **Class** | **% probability** |
| 127 | OC_022_D_CT_5 | Cut | Peritoneum | ?normal | Normal |  | Fibrous tissue | Normal |  | 6.8 | Normal | 98.0% |
| 128 | OC_022_D_CT_6 | Cut | Peritoneum | ?normal | Normal |  | Fibrous tissue | Normal |  | 3.5 | Normal | 97.8% |
| 129 | OC_022_D_CT_7 | Cut | Peritoneum | ?normal | Normal |  | Fibrous tissue | Normal |  | 5.6 | Normal | 96.7% |
| 130 | OC_022_D_CT_8 | Cut | Peritoneum | ?normal | Normal |  | Fibrous tissue | Normal |  | 1.5 | Normal | 95.6% |
| 131 | OC_022_E_CG_1 | Coagulation | Omentum | ?tumour | Cancer |  | Carcinosarcoma | Cancer | 90 |  | Cancer | 97.2% |
| 132 | OC_022_E_CG_2 | Coagulation | Omentum | ?tumour | Cancer |  | Carcinosarcoma | Cancer | 50 |  | Cancer | 97.6% |
| 133 | OC_022_E_CG_3 | Coagulation | Omentum | ?tumour | Cancer |  | Carcinosarcoma | Cancer | 50 |  | Normal | 87.5% |
| 134 | OC_022_E_CG_4 | Coagulation | Omentum | ?tumour | Cancer |  | Carcinosarcoma | Cancer | 90 |  | Cancer | 61.4% |
| 135 | OC_022_E_CG_5 | Coagulation | Omentum | ?tumour | Cancer |  | Carcinosarcoma | Cancer | 30 |  | Normal | 80.3% |
| 136 | OC_022_E_CG_6 | Coagulation | Omentum | ?normal | Normal |  | Adipose tissue | Normal |  | 6.5 | Normal | 95.6% |
| 137 | OC_022_E_CG_7 | Coagulation | Omentum | ?normal | Normal |  | Adipose tissue | Normal |  | 11.4 | Normal | 92.9% |
| 138 | OC_022_E_CG_8 | Coagulation | Omentum | ?normal | Normal |  | Adipose tissue | Normal |  | 7.5 | Normal | 92.9% |
| 139 | OC_022_E_CG_9 | Coagulation | Omentum | ?normal | Normal |  | Adipose tissue | Normal |  | 10.1 | Normal | 93.0% |
| 140 | OC_022_E_CG_10 | Coagulation | Omentum | ?normal | Normal |  | Adipose tissue | Normal |  | 8.9 | Normal | 92.0% |
| 141 | OC_022_E_CT_1 | Cut | Omentum | ?tumour | Cancer |  | Carcinosarcoma | Cancer | 30 |  | Normal | 84.0% |
| 142 | OC_022_E_CT_2 | Cut | Omentum | ?tumour | Cancer |  | Adipose tissue | Normal |  | 1.3 | Normal | 86.0% |
| 143 | OC_022_E_CT_3 | Cut | Omentum | ?tumour | Cancer |  | Carcinosarcoma | Cancer | 60 |  | Normal | 68.1% |
| 144 | OC_022_E_CT_4 | Cut | Omentum | ?tumour | Cancer |  | Fibrous tissue + adipose tissue + lymphocytes | Normal |  | 3.2 | Normal | 79.5% |
| 145 | OC_022_E_CT_5 | Cut | Omentum | ?tumour | Cancer |  | Fibrous tissue + adipose tissue | Normal |  | 3.0 | Normal | 54.8% |
| 146 | OC_022_E_CT_6 | Cut | Omentum | ?normal | Normal |  | Adipose tissue | Normal |  | 8.9 | Normal | 79.8% |
| 147 | OC_022_E_CT_7 | Cut | Omentum | ?normal | Normal |  | Adipose tissue | Normal |  | 5.0 | Normal | 85.3% |
| 148 | OC_022_E_CT_8 | Cut | Omentum | ?normal | Normal |  | Adipose tissue | Normal |  | 6.0 | Normal | 89.5% |
| 149 | OC_022_E_CT_9 | Cut | Omentum | ?normal | Normal |  | Adipose tissue | Normal |  | 3.7 | Normal | 81.5% |
| 150 | OC_022_E_CT_10 | Cut | Omentum | ?normal | Normal |  | Adipose tissue | Normal |  | 4.1 | Normal | 88.6% |
