# Supplementary material for: The surgical intelligent knife distinguishes normal, borderline and malignant gynaecological tissues using rapid evaporative ionisation mass spectrometry (REIMS)
Source: Br J Cancer. 2018 Apr 19;118(10):1349–58. doi: 10.1038/s41416-018-0048-3 (PMC5959892; doi:10.1038/s41416-018-0048-3)
Supplement: Supplementary file 7 — Supplementary Table 6 [file 41416_2018_48_MOESM7_ESM.docx]

| ***m/z***  **feature** | **MannU**  ***P*** | **MannU**  **q** | **Normal (mean a.i.)** | **Normal (median a.i.)** | **Cancer (mean a.i.)** | **Cancer (median a.i.)** | **Median log2 FC Normal vs Cancer** |
| --- | --- | --- | --- | --- | --- | --- | --- |
| 600.5 | 1.23E-19 | 3.25E-19 | 11.26 | 10.91 | 5.59 | 5.51 | 0.98 |
| 601.5 | 4.22E-15 | 6.89E-15 | 4.91 | 4.59 | 2.95 | 2.72 | 0.76 |
| 602.3 | 4.95E-16 | 8.40E-16 | 3.70 | 3.40 | 1.90 | 1.53 | 1.15 |
| 602.5 | 3.02E-16 | 5.27E-16 | 4.07 | 3.93 | 2.12 | 2.08 | 0.91 |
| 603.5 | 6.89E-10 | 8.46E-10 | 4.92 | 4.03 | 3.08 | 2.93 | 0.46 |
| 628.5 | 1.02E-07 | 1.11E-07 | 6.29 | 5.96 | 4.79 | 4.64 | 0.36 |
| 629.5 | 1.47E-07 | 1.59E-07 | 17.49 | 15.50 | 12.37 | 13.22 | 0.23 |
| 645.4 | 4.77E-29 | 3.11E-28 | 2.04 | 2.03 | 4.45 | 3.78 | -0.90 |
| 645.5 | 7.47E-29 | 4.21E-28 | 4.99 | 4.60 | 9.95 | 8.71 | -0.92 |
| 647.5 | 9.79E-12 | 1.33E-11 | 2.56 | 1.49 | 3.86 | 3.34 | -1.16 |
| 656.6 | 9.11E-14 | 1.41E-13 | 6.19 | 5.78 | 3.99 | 3.41 | 0.76 |
| 657.5 | 3.78E-08 | 4.26E-08 | 26.52 | 25.06 | 19.71 | 18.73 | 0.42 |
| 657.6 | 1.79E-18 | 4.11E-18 | 4.99 | 4.98 | 2.92 | 2.60 | 0.94 |
| 658.5 | 8.84E-07 | 8.99E-07 | 10.97 | 11.09 | 8.70 | 8.39 | 0.40 |
| 658.6 | 1.86E-07 | 1.96E-07 | 3.21 | 3.27 | 2.40 | 2.12 | 0.63 |
| 663.5 | 8.05E-22 | 2.49E-21 | 3.74 | 3.42 | 0.87 | 0.40 | 3.09 |
| 670.5 | 7.65E-23 | 2.64E-22 | 5.99 | 5.70 | 2.66 | 1.70 | 1.74 |
| 671.4 | 4.78E-24 | 1.80E-23 | 2.64 | 2.52 | 4.82 | 4.55 | -0.85 |
| 671.5 | 6.79E-28 | 3.37E-27 | 12.87 | 11.66 | 25.15 | 23.40 | -1.00 |
| 672.5 | 6.37E-35 | 1.32E-33 | 3.17 | 2.58 | 9.62 | 8.92 | -1.79 |
| 673.5 | 2.23E-19 | 5.65E-19 | 38.51 | 32.24 | 56.61 | 52.55 | -0.70 |
| 674.5 | 1.55E-20 | 4.36E-20 | 13.02 | 11.03 | 20.66 | 19.82 | -0.85 |
| 675.5 | 6.16E-16 | 1.03E-15 | 3.38 | 2.78 | 5.44 | 5.39 | -0.96 |
| 679.5 | 5.85E-18 | 1.27E-17 | 18.96 | 18.44 | 10.29 | 8.69 | 1.09 |
| 680.5 | 2.27E-11 | 2.97E-11 | 6.37 | 5.89 | 3.28 | 2.26 | 1.38 |
| 681.5 | 1.73E-08 | 2.01E-08 | 6.97 | 6.14 | 4.76 | 3.28 | 0.91 |
| 682.6 | 5.58E-18 | 1.24E-17 | 22.19 | 20.47 | 11.75 | 10.27 | 0.99 |
| 683.5 | 6.30E-13 | 9.19E-13 | 1.80 | 1.12 | 4.23 | 3.33 | -1.57 |
| 683.6 | 6.18E-13 | 9.12E-13 | 10.17 | 8.92 | 5.92 | 5.39 | 0.73 |
| 685.5 | 2.10E-12 | 2.99E-12 | 15.52 | 14.80 | 21.56 | 19.67 | -0.41 |
| 686.5 | 2.50E-10 | 3.14E-10 | 3.28 | 2.83 | 5.46 | 5.00 | -0.82 |
| 687.6 | 2.40E-06 | 2.40E-06 | 24.90 | 24.19 | 20.23 | 19.17 | 0.34 |
| 691.5 | 3.89E-16 | 6.69E-16 | 8.57 | 8.61 | 4.84 | 4.03 | 1.09 |
| 695.5 | 2.54E-23 | 9.00E-23 | 2.63 | 2.28 | 5.63 | 5.62 | -1.30 |
| 697.5 | 5.39E-31 | 5.57E-30 | 27.08 | 23.45 | 59.10 | 55.89 | -1.25 |
| 698.5 | 7.04E-29 | 4.16E-28 | 10.60 | 8.55 | 27.44 | 26.44 | -1.63 |
| 699.5 | 2.01E-34 | 3.12E-33 | 72.62 | 68.47 | 136.41 | 130.31 | -0.93 |
| 700.5 | 1.71E-37 | 7.08E-36 | 32.57 | 30.49 | 63.65 | 61.81 | -1.02 |
| 700.6 | 2.01E-09 | 2.44E-09 | 3.09 | 3.09 | 4.63 | 4.09 | -0.40 |
| 701.5 | 1.08E-10 | 1.38E-10 | 33.89 | 29.33 | 44.23 | 42.83 | -0.55 |
| 702.5 | 1.07E-11 | 1.45E-11 | 9.55 | 7.99 | 13.34 | 12.31 | -0.62 |
| 707.5 | 2.47E-09 | 2.97E-09 | 6.13 | 5.77 | 3.78 | 2.64 | 1.13 |
| 711.5 | 6.27E-24 | 2.29E-23 | 4.61 | 3.99 | 9.28 | 9.08 | -1.19 |
| 713.5 | 6.70E-17 | 1.32E-16 | 9.57 | 9.15 | 15.28 | 14.44 | -0.66 |
| 714.5 | 5.88E-32 | 6.63E-31 | 1.89 | 0.91 | 8.31 | 6.62 | -2.86 |
| 715.5 | 4.03E-18 | 9.08E-18 | 35.92 | 36.03 | 24.36 | 23.04 | 0.64 |
| 715.6 | 1.41E-38 | 8.71E-37 | 13.90 | 13.97 | 5.53 | 4.67 | 1.58 |
| 716.6 | 7.25E-17 | 1.38E-16 | 7.14 | 6.86 | 4.61 | 4.28 | 0.68 |
| 717.5 | 3.15E-08 | 3.62E-08 | 37.31 | 37.32 | 29.95 | 27.43 | 0.44 |
| 719.5 | 3.07E-17 | 6.24E-17 | 9.48 | 9.37 | 4.95 | 4.08 | 1.20 |
| 723.5 | 7.33E-17 | 1.38E-16 | 34.92 | 31.27 | 51.46 | 49.59 | -0.67 |
| 724.5 | 5.96E-21 | 1.80E-20 | 8.66 | 7.35 | 17.22 | 16.28 | -1.15 |
| 725.5 | 1.23E-36 | 3.83E-35 | 22.84 | 21.08 | 51.43 | 48.23 | -1.19 |
| 726.5 | 1.24E-30 | 1.10E-29 | 9.53 | 8.28 | 21.70 | 20.06 | -1.28 |
| 727.5 | 1.71E-29 | 1.18E-28 | 10.90 | 9.95 | 24.48 | 22.93 | -1.20 |
| 727.6 | 2.87E-12 | 4.05E-12 | 6.45 | 6.07 | 8.59 | 8.30 | -0.45 |
| 728.5 | 6.29E-31 | 6.00E-30 | 3.69 | 3.50 | 9.25 | 8.30 | -1.25 |
| 728.6 | 3.06E-19 | 7.59E-19 | 6.55 | 6.33 | 10.82 | 9.99 | -0.66 |
| 729.6 | 3.47E-20 | 9.57E-20 | 1.80 | 1.62 | 4.03 | 3.21 | -0.99 |
| 739.5 | 1.38E-18 | 3.23E-18 | 11.96 | 11.65 | 6.65 | 5.97 | 0.96 |
| 740.5 | 7.99E-14 | 1.25E-13 | 2.79 | 2.42 | 7.38 | 5.38 | -1.15 |
| 742.5 | 1.15E-22 | 3.86E-22 | 28.20 | 27.70 | 46.72 | 42.91 | -0.63 |
| 742.6 | 5.45E-22 | 1.73E-21 | 16.17 | 15.55 | 25.63 | 23.59 | -0.60 |
| 743.5 | 1.66E-08 | 1.94E-08 | 19.79 | 18.43 | 25.06 | 23.86 | -0.37 |
| 744.5 | 3.17E-13 | 4.79E-13 | 37.26 | 37.04 | 46.71 | 45.79 | -0.31 |
| 744.6 | 2.73E-16 | 4.84E-16 | 60.33 | 59.75 | 77.34 | 74.19 | -0.31 |
| 745.5 | 6.00E-13 | 8.96E-13 | 17.56 | 17.47 | 21.83 | 20.98 | -0.26 |
| 745.6 | 1.45E-11 | 1.94E-11 | 27.62 | 26.94 | 34.68 | 32.82 | -0.28 |
| 746.5 | 3.45E-08 | 3.92E-08 | 4.16 | 4.16 | 6.99 | 5.42 | -0.38 |
| 746.6 | 9.64E-08 | 1.06E-07 | 8.35 | 8.40 | 10.47 | 10.61 | -0.34 |
| 747.5 | 6.53E-39 | 8.10E-37 | 10.56 | 10.27 | 31.80 | 26.57 | -1.37 |
| 750.6 | 5.30E-12 | 7.30E-12 | 21.00 | 21.81 | 14.54 | 13.09 | 0.74 |
| 751.6 | 1.72E-11 | 2.26E-11 | 9.96 | 9.82 | 6.79 | 6.10 | 0.69 |
| 752.6 | 1.89E-13 | 2.90E-13 | 24.72 | 24.19 | 19.38 | 18.18 | 0.41 |
| 753.6 | 3.02E-09 | 3.60E-09 | 9.14 | 8.95 | 7.29 | 6.53 | 0.45 |
| 754.6 | 5.47E-07 | 5.61E-07 | 10.95 | 10.92 | 15.13 | 14.99 | -0.46 |
| 755.6 | 2.80E-07 | 2.92E-07 | 3.59 | 3.38 | 5.61 | 5.43 | -0.68 |
| 756.6 | 3.47E-20 | 9.36E-20 | 2.00 | 2.00 | 4.92 | 4.54 | -1.18 |
| 759.5 | 1.80E-07 | 1.92E-07 | 3.59 | 2.93 | 5.03 | 5.00 | -0.77 |
| 761.5 | 5.32E-07 | 5.50E-07 | 7.09 | 6.39 | 9.65 | 9.18 | -0.52 |
| 764.5 | 6.85E-17 | 1.33E-16 | 7.49 | 6.65 | 11.76 | 11.86 | -0.84 |
| 768.5 | 1.09E-17 | 2.26E-17 | 12.45 | 12.45 | 17.35 | 16.92 | -0.44 |
| 768.6 | 1.55E-19 | 4.01E-19 | 16.18 | 16.08 | 23.76 | 22.61 | -0.49 |
| 769.5 | 5.92E-15 | 9.54E-15 | 8.99 | 8.98 | 12.88 | 12.18 | -0.44 |
| 769.6 | 3.81E-11 | 4.92E-11 | 8.77 | 8.69 | 11.55 | 11.11 | -0.36 |
| 770.5 | 3.48E-17 | 6.95E-17 | 6.49 | 6.36 | 9.16 | 8.83 | -0.47 |
| 770.6 | 8.78E-17 | 1.60E-16 | 30.96 | 30.82 | 40.88 | 40.73 | -0.40 |
| 771.5 | 3.34E-36 | 8.28E-35 | 4.37 | 3.96 | 12.16 | 11.57 | -1.55 |
| 772.5 | 4.89E-25 | 2.02E-24 | 1.77 | 1.55 | 4.02 | 3.51 | -1.18 |
| 773.5 | 1.03E-34 | 1.82E-33 | 2.98 | 1.74 | 9.02 | 8.30 | -2.25 |
| 773.6 | 7.66E-21 | 2.21E-20 | 7.06 | 6.76 | 10.74 | 9.96 | -0.56 |
| 776.5 | 1.10E-16 | 1.97E-16 | 6.60 | 6.28 | 4.55 | 4.09 | 0.62 |
| 776.6 | 6.27E-18 | 1.34E-17 | 4.66 | 4.50 | 3.13 | 2.78 | 0.69 |
| 778.6 | 1.03E-12 | 1.49E-12 | 5.88 | 6.00 | 4.14 | 3.71 | 0.69 |
| 789.5 | 6.97E-08 | 7.72E-08 | 5.73 | 4.63 | 7.76 | 8.01 | -0.79 |
| 790.5 | 9.70E-15 | 1.54E-14 | 8.05 | 8.18 | 11.12 | 10.77 | -0.40 |
| 792.5 | 1.84E-07 | 1.95E-07 | 9.86 | 9.81 | 11.75 | 11.88 | -0.28 |
| 794.5 | 4.52E-12 | 6.30E-12 | 6.65 | 6.30 | 8.74 | 8.64 | -0.46 |
| 795.5 | 2.15E-25 | 9.21E-25 | 2.85 | 2.64 | 5.03 | 4.96 | -0.91 |
| 797.6 | 4.08E-10 | 5.06E-10 | 12.86 | 12.16 | 9.99 | 9.16 | 0.41 |
| 797.7 | 9.27E-27 | 4.42E-26 | 15.09 | 14.27 | 8.41 | 7.28 | 0.97 |
| 798.7 | 8.40E-25 | 3.36E-24 | 8.15 | 7.64 | 4.71 | 4.10 | 0.90 |
| 804.6 | 8.79E-30 | 6.41E-29 | 3.21 | 3.15 | 1.20 | 1.01 | 1.64 |
| 816.5 | 1.34E-08 | 1.59E-08 | 2.76 | 2.66 | 3.44 | 3.48 | -0.39 |
| 816.6 | 2.63E-26 | 1.21E-25 | 5.91 | 5.98 | 3.51 | 3.41 | 0.81 |
| 817.6 | 6.67E-21 | 1.97E-20 | 4.42 | 4.52 | 2.50 | 2.31 | 0.97 |
| 818.5 | 4.99E-29 | 3.09E-28 | 3.07 | 2.85 | 5.60 | 5.72 | -1.01 |
| 818.6 | 8.78E-18 | 1.85E-17 | 11.11 | 10.92 | 7.74 | 7.52 | 0.54 |
| 819.5 | 2.06E-25 | 9.14E-25 | 2.77 | 2.41 | 5.68 | 4.93 | -1.03 |
| 819.6 | 8.35E-16 | 1.38E-15 | 5.81 | 5.83 | 3.94 | 3.60 | 0.69 |
| 820.5 | 5.86E-28 | 3.03E-27 | 1.82 | 1.65 | 3.72 | 3.50 | -1.09 |
| 822.6 | 1.64E-30 | 1.35E-29 | 2.13 | 2.03 | 4.31 | 4.38 | -1.11 |
| 832.6 | 1.88E-10 | 2.38E-10 | 3.51 | 3.47 | 2.59 | 2.53 | 0.46 |
| 833.6 | 6.47E-08 | 7.23E-08 | 3.49 | 3.45 | 2.69 | 2.72 | 0.35 |
| 844.6 | 1.90E-06 | 1.91E-06 | 4.30 | 4.31 | 3.53 | 3.56 | 0.28 |
| 845.6 | 3.40E-24 | 1.32E-23 | 3.59 | 3.57 | 2.07 | 2.08 | 0.78 |
| 848.6 | 6.01E-34 | 8.27E-33 | 2.96 | 2.88 | 5.28 | 5.11 | -0.83 |
| 849.6 | 3.16E-32 | 3.92E-31 | 1.73 | 1.68 | 3.39 | 3.26 | -0.95 |
| 862.6 | 1.48E-28 | 8.00E-28 | 2.64 | 2.52 | 4.28 | 4.06 | -0.69 |
| 863.6 | 4.79E-30 | 3.71E-29 | 1.86 | 1.74 | 4.49 | 3.83 | -1.13 |
| 887.5 | 5.58E-19 | 1.33E-18 | 3.02 | 3.03 | 4.82 | 4.41 | -0.54 |
| 887.6 | 8.58E-17 | 1.59E-16 | 5.43 | 4.99 | 9.97 | 9.51 | -0.93 |
| 888.6 | 3.94E-19 | 9.58E-19 | 2.48 | 2.31 | 4.63 | 4.31 | -0.90 |
| 893.7 | 2.60E-22 | 8.47E-22 | 16.05 | 3.81 | 1.24 | 0.59 | 2.69 |

**Supplementary Table 6:** All 124 *m/z* peaks with q<0.001 contributing to class separation in OC vs Normal tissue model. a.i.: arbitrary intensity, Mann U: Mann Whitney U Test, FC: fold change
