# Supplementary material for: The surgical intelligent knife distinguishes normal, borderline and malignant gynaecological tissues using rapid evaporative ionisation mass spectrometry (REIMS)
Source: Br J Cancer. 2018 Apr 19;118(10):1349–58. doi: 10.1038/s41416-018-0048-3 (PMC5959892; doi:10.1038/s41416-018-0048-3)
Supplement: Supplementary file 8 — Supplementary Table 7 [file 41416_2018_48_MOESM8_ESM.docx]

| ***m/z***  **feature** | **MannU**  ***P*** | **MannU**  **q** | **Normal (mean a.i.)** | **Normal (median a.i.)** | **BOT**  **(mean a.i.)** | **BOT (median a.i.)** | **Median log 2 FC Normal vs BOT** |
| --- | --- | --- | --- | --- | --- | --- | --- |
| 600.5 | 2.86E-07 | 3.87E-07 | 8.37 | 8.44 | 5.56 | 5.47 | 0.63 |
| 645.4 | 4.55E-11 | 3.07E-10 | 2.27 | 2.26 | 4.42 | 3.77 | -0.74 |
| 645.5 | 3.29E-07 | 4.04E-07 | 6.25 | 5.83 | 9.85 | 8.56 | -0.55 |
| 656.6 | 4.41E-08 | 7.43E-08 | 5.72 | 5.38 | 3.94 | 3.38 | 0.67 |
| 657.6 | 2.64E-10 | 1.19E-09 | 5.22 | 4.78 | 2.91 | 2.62 | 0.87 |
| 658.6 | 4.79E-07 | 4.97E-07 | 3.63 | 3.65 | 2.38 | 2.07 | 0.82 |
| 671.4 | 1.15E-13 | 1.55E-12 | 2.31 | 2.29 | 4.82 | 4.54 | -0.99 |
| 671.5 | 5.82E-13 | 5.24E-12 | 12.40 | 12.20 | 25.10 | 23.56 | -0.95 |
| 672.5 | 4.03E-15 | 1.09E-13 | 3.12 | 2.91 | 9.60 | 8.76 | -1.59 |
| 679.5 | 3.71E-07 | 4.35E-07 | 18.36 | 18.74 | 10.37 | 8.98 | 1.06 |
| 682.6 | 6.97E-11 | 3.77E-10 | 22.45 | 19.18 | 11.62 | 9.82 | 0.96 |
| 683.6 | 1.03E-09 | 3.48E-09 | 10.58 | 9.38 | 5.87 | 5.36 | 0.81 |
| 685.5 | 3.34E-08 | 6.01E-08 | 13.85 | 11.88 | 21.68 | 19.70 | -0.73 |
| 686.5 | 4.83E-10 | 1.86E-09 | 2.21 | 1.91 | 5.50 | 5.08 | -1.41 |
| 695.5 | 7.91E-09 | 1.94E-08 | 2.80 | 2.28 | 5.64 | 5.65 | -1.31 |
| 697.5 | 2.17E-08 | 4.19E-08 | 33.08 | 27.48 | 59.85 | 55.98 | -1.03 |
| 698.5 | 1.24E-09 | 3.73E-09 | 11.89 | 8.83 | 27.76 | 26.45 | -1.58 |
| 699.5 | 4.01E-07 | 4.52E-07 | 98.17 | 92.51 | 137.59 | 130.45 | -0.50 |
| 700.5 | 1.99E-08 | 4.14E-08 | 43.21 | 40.24 | 64.16 | 62.35 | -0.63 |
| 719.6 | 5.93E-07 | 5.93E-07 | 4.07 | 4.48 | 2.03 | 1.66 | 1.43 |
| 740.5 | 3.76E-09 | 1.02E-08 | 1.86 | 1.65 | 7.25 | 5.23 | -1.66 |
| 742.5 | 1.22E-07 | 1.84E-07 | 30.18 | 27.87 | 47.07 | 42.94 | -0.62 |
| 742.6 | 4.09E-07 | 4.42E-07 | 17.50 | 16.08 | 25.85 | 24.02 | -0.58 |
| 773.5 | 3.04E-07 | 3.91E-07 | 5.04 | 4.73 | 8.91 | 8.07 | -0.77 |
| 785.5 | 9.96E-08 | 1.58E-07 | 7.86 | 7.46 | 4.12 | 3.31 | 1.17 |
| 845.6 | 8.09E-09 | 1.82E-08 | 3.13 | 3.17 | 2.06 | 2.08 | 0.61 |
| 888.6 | 1.38E-07 | 1.97E-07 | 2.52 | 2.28 | 4.59 | 4.29 | -0.91 |

**Supplementary Table 7:** All 27 *m/z* peaks with q<0.001 contributing to class separation in OC vs Borderline tissue model. a.i.: arbitrary intensity, Mann U: Mann Whitney U Test, BOT: borderline ovarian tumour, FC: fold change
